# ABHD17C, a metabolic and immune-related gene signature, predicts prognosis and anti-PD1 therapy response in pancreatic cancer

**DOI:** 10.1007/s12672-023-00690-7

**Published:** 2023-06-05

**Authors:** Weihao Zhang, Yongjie Xie, Xin Yu, Changfu Liu, Wei Gao, Wenge Xing, Tongguo Si

**Affiliations:** 1 Tianjin Medical University Cancer Institute & Hospital, National Clinical Research Center for Cancer, Tianjin, 300060 People’s Republic of China; 2 Tianjin’s Clinical Research Center for Cancer, Tianjin, China; 3 Key Laboratory of Cancer Prevention and Therapy, Tianjin, China; 4 Department of Pancreatic Cancer, Key Laboratory of Cancer Prevention and Therapy, Tianjin Medical University Cancer Institute and Hospital, National Clinical Research Center for Cancer, Tianjin, China

**Keywords:** Gene signature, Metabolism, ABHD17C, Pancreatic cancer, Glycolysis, Immune microenvironment

## Abstract

**Background:**

PDAC is a highly malignant and immune-suppressive tumor, posing great challenges to therapy.

**Methods:**

In this study, we utilized multi-center RNA sequencing and non-negative matrix factorization clustering (NMF) to identify a group of metabolism-related genes that could effectively predict the immune status and survival (both disease-free survival and overall survival) of pancreatic ductal adenocarcinoma (PDAC) patients. Subsequently, through the integration of single cell sequencing and our center's prospective and retrospective cohort studies, we identified ABHD17C, which possesses metabolic and immune-related characteristics, as a potential biomarker for predicting the prognosis and response to anti-PD1 therapy in PDAC. We then demonstrated how ABHD17C participates in the regulation of the immune microenvironment through in vitro glycolytic function experiments and in vivo animal experiments.

**Results:**

Through screening for pancreatic cancer metabolic markers and immune status, we identified a critical molecule that inhibits pancreatic cancer survival and prognosis. Further flow cytometry analysis confirmed that ABHD17C is involved in the inhibition of the formation of the immune environment in PDAC. Our research found that ABHD17C participates in the metabolic process of tumor cells in in vitro and in vivo experiments, reshaping the immunosuppressive microenvironment by downregulating the pH value. Furthermore, through LDHA inhibition experiments, we demonstrated that ABHD17C significantly enhances glycolysis and inhibits the formation of the immune suppressive environment. In in vivo experiments, we also validated that ABHD17C overexpression significantly mediates resistance to anti-PD1 therapy and promotes the progression of pancreatic cancer.

**Conclusion:**

Therefore, ABHD17C may be a novel and effective biomarker for predicting the metabolic status and immune condition of PDAC patients, and provide a potential predictive strategy for anti-PD1 therapy in PDAC.

**Supplementary Information:**

The online version contains supplementary material available at 10.1007/s12672-023-00690-7.

## Introduction

Pancreatic cancer is a malignant tumor of the digestive tract with hidden and atypical clinical symptoms, which is difficult to diagnose and treat [1]. According to The Lancet, the five-year survival rate of pancreatic cancer after diagnosis is about 10%, which is one of the malignant tumors with the worst prognosis [2]. About 90% of pancreatic cancer is ductal adenocarcinoma originating from adenocarcinoma epithelium [3]. Its incidence rate and mortality have increased significantly in recent years. The early diagnosis rate of pancreatic cancer is not high, the operative mortality rate is high, and the cure rate is low [4]. Available treatment strategies for patients with PDAC include chemotherapy, surgical resection, radiotherapy, and immunotherapy [5]. Common PDAC chemotherapy drugs include Fluorouracil, Cisplatin, Gemcitabine, and Paclitaxel [6]. Survival for patients with PDAC depends largely on age at diagnosis, disease stage, and PDAC biology. Especially in high-risk patients, disease prognosis remains poor and recurrence rates are high [7]. The existence of this condition underscores the need to develop new therapeutic strategies and treatments [8].

Metabolism is the basis of all biological processes necessary to sustain life. It involves a series of biochemical reactions that convert nutrients into small molecules called metabolites [9]. Through these transformations and the resulting metabolites, cells produce the energy, redox equivalents, and macromolecules (including proteins, lipids, DNA, and RNA) they need to survive and maintain cellular function [10]. It is well known that under aerobic conditions, normal cells first undergo glycolysis in the cytosol followed by mitochondrial oxidative phosphorylation (OXPHOS) to obtain energy [11]. When hypoxia, cells rely on glycolysis rather than oxygen-consuming mitochondrial metabolism for energy [12]. However, the metabolic pattern of tumors differs from that of normal cells [13]. As first observed by Otto Warburg, the phenomenon that cancer cells prefer to undergo glycolysis in the cytosol even in the presence of oxygen is known as the “Warburg effect" or "aerobic glycolysis” [14]. Because the immortal proliferation of tumor cells requires a faster energy supply, the rate of ATP production by glycolysis is much faster than that by oxidative phosphorylation, although the production of ATP per glucose molecule is much less efficient[15]. Current studies have found that metabolism-related genes are closely related to the occurrence, development, and prognosis of tumors [16]. Cancer cells undergo a process of gradual adaptation to metabolism, which allows tumor cells to grow and proliferate rapidly, thereby supporting tumor initiation and progression. Accumulating evidence points to the fact that immune responses are associated with marked alterations in tissue metabolism, including nutrient consumption, increased oxygen consumption, and production of reactive nitrogen and oxygen intermediates [17]. Likewise, many metabolites in the tumor microenvironment also affect immune cell differentiation and effector function [18]. But recent studies have shown that immune cells compete with cancer cells and other proliferating cells for nutrients in the microenvironment [19]. Thus, it suggests that metabolic intervention holds promise for improving the effectiveness of immunotherapy.

In the present study, information on genes related to metabolism was collected. Gene expression data from multi-center platforms were used to construct PDAC molecular subtypes based on genes related to energy metabolism. The relationship between molecular subtypes and prognosis was further evaluated. Finally, ABHD17C was screened as an independent prognostic evaluation index for PDAC patients with LASSO-Cox regression analysis. The marker can evaluate the prognosis of PC patients and be validated in vivo and in vitro assays. In addition, we evaluated the function of ABHD17C in terms of clinical relevance, metabolic relevance, immune landscape, and prediction of anti-PD1 therapy.

## Materials and methods

### Data acquisition

Three separate PDAC cohorts with intact genetic expression profiles and clinical information were enrolled in our work: The Cancer Genome Atlas (TCGA, https://portal.gdc. cancer.gov/) cohort; the GSE62452 (GEO, https://www.ncbi.nlm.nih.gov/geo/) cohort; The clinicopathologic data for all cohorts are displayed in Supplementary table 5. The TCGA public database that we downloaded contains mainly three types of pancreatic cancer data: Pancreas-Adenocarcinoma Ductal Type (150), Pancreas-Adenocarcinoma-Other Subtype [24], and Pancreas-Colloid (mucinous non-cystic) Carcinoma [4]. The raw single cell RNA sequence data presented in this paper have been deposited in the Genome Sequence Archive (CRA001160) at the National Genomics Data Center (NGDC) of the Chinese Academy of Sciences. We derived metabolism-related gene sets from classical gene sets available in the GSEA database. Genetic expression profiles and clinical information of six PDAC patients in our center were listed in Supplementary table 1. We adhered strictly to the rules of accessing the publicly available database, and since the data was obtained from a public database, we did not require approval from the regional ethics board. All patients provided written informed consent for the use of their specimens and disease information for future research following the Ethics Committee of Tianjin Medical University Cancer Institute and Hospital, China, and under the tenets of the Declaration of Helsinki (ID number of ethics approval: PMIF-2021014).

### Comprehensive analysis of immune infiltration characteristics of different metabolism-related subgroups

Based on the RNA-seq dataset of GSE62452 database (GEO), we evaluated the immune infiltration characteristics of PDAC by seven online tools: CIBERSORT, TIMER, CIBERSORT_ABS, EPIC, MCPcounter, Quantiseq. Then, we compared the immune cells in the six genes which are consistent with our prognostic signature.

### Energy metabolic molecular subtypes

We analyzed the metabolism-related molecular subtype of PDAC using a total of 594 genes. We utilized non-negative matrix factorization (NMF) consensus clustering from the "NMF" R package to cluster all PDAC samples in the GSE62452 dataset. We performed survival analysis and independence tests on the clustering results. We compared the gene set variation analysis (GSVA) of different groups and obtained the immune scores of the subtypes through the use of the TIMER (tumor immune estimation resource) tool.

### DEGs identification and bioinformatics analysis

We employed the "DESeq2" R package to compute the differentially expressed genes (DEGs) of the subtypes with a false discovery rate (FDR) < 0.05 and absolute log2 fold change (|log2FC|) > 1. Subsequently, we conducted Gene Ontology (GO) and Kyoto Encyclopedia of Genes and Genomes (KEGG) functional enrichment analyses based on these DEGs. The differentially expressed genes (DEGs) were listed in Supplementary table 2.

### Western blot analysis

For protein isolation, either pancreatic cancer tissue samples or pancreatic cancer cell lines were used, and the obtained protein was processed for western blotting. Equal amounts of protein were loaded onto SDS-PAGE gels and transferred to PVDF membranes. The membranes were blocked with 5% nonfat dry milk diluted in TBST for 1 h, and incubated with primary antibodies against GLUT1(ab15309, Abcam, UK), GLUT4(ab33780, Abcam, UK), MCT1 (EMD Millipore Corporation, USA), MCT4(sc-50329, SCB, USA), ABHD17C(PA5-61831, Thermofisher) overnight at 4 °C. The following day, the blots were incubated with the appropriate secondary antibody (1:5000) for 1 h at room temperature. The blots were then visualized using an ECL kit (eBioscience), and imaged using an Image Lab imaging system. The amount of protein loaded was 50 μg for cell culture and 100 μg for patient tissue.

### Immunocytochemistry staining

Immunohistochemistry (IHC) was employed to detect ABHD17C in tumour tissue. Firstly, paraffin-embedded sections of tumour tissue were deparaffinized and subjected to antigen retrieval by heat treatment in a pressure cooker for 3 min. The sections were then incubated with primary antibodies overnight at 4 °C. Subsequently, a peroxidase-conjugated secondary antibody was used to detect antibody binding at 37 °C for 30 min. A chromogenic reaction was carried out using a DAB substrate kit. Staining intensity was evaluated using a scale of 0 (negative), 1 (low), 2 (medium), or 3 (high), while the extent of staining was scored as 0 (0% stained), 1 (1–25% stained), 2 (26–50% stained), or 3 (51–100% stained). Five random fields (20 × magnification) were evaluated using a light microscope. The final staining score was determined by multiplying the intensity and extent scores and dividing the samples into four grades: 0 (negative, −), 1–2 (low staining, +), 3–5 (medium staining, + +), and 6–9 (high staining, +  + +).

### Construction and validation of risk model

The training cohort's expression data of the DEGs were utilized to construct a risk score model. The impact of each DEG on the overall survival (OS) of PDAC patients was estimated using the univariate Cox proportional risk regression model. Statistical significance was considered at Log-rank P < 0.01. To narrow down the number of genes in our model, we employed LASSO-Cox regression. The risk score model included individual normalized gene expression values weighted by their LASSO-Cox coefficients. We validated the robustness of the risk model using internal and external validation cohorts. The R package "timeROC" was used to plot the risk score distribution of each sample. Subsequently, we used the Gordon index to calculate the cutoff value and divide the samples into high- and low-risk groups. We compared the survival difference between the two groups using a log-rank test and performed the K-M survival curve to analyze the OS of each group.

### Prognostic value of the risk signature in training and validation group

The patients were classified into high- and low-risk groups based on the median value of the risk score, and the prognostic ability of the risk signature was demonstrated by constructing Kaplan–Meier (K-M) survival curves with the Log-rank test. Moreover, we evaluated the performance of the two signatures by calculating the area under the curve values (AUCs) of the receiver operating characteristic (ROC) curves for 1-, 2-, 3-, and 5-year survival using the R package "survivalROC."

### Cell culture

The human pancreatic ductal adenocarcinoma (PDAC) cell line, PANC-1, was obtained from the Type Culture Collection Committee of the Chinese Academy of Sciences (Shanghai, China) in 2013. The murine pancreatic ductal adenocarcinoma (PDAC) cell line derived from KPC mouse was generously provided by Dr. Tingbo Liang from the Department of Surgery at the First Affiliated Hospital of Zhejiang University. Additionally, ABHD17C-overexpressing cell lines were constructed in these two cell lines. Cell suspensions that meet the requirements of cell counting were divided into culture flasks, and the culture flasks were placed in a culture box at 37 °C and 5% CO2 for 24 h, and the medium was changed to continue the culture. The optimal cell concentration was 5 × 105/ml. The preparation method of cell suspension is to digest with 0.25% trypsin solution, wash with PBS solution, add culture solution (or Hanks solution or balanced salt solution such as PBS), and blow to prepare the cell suspension to be tested. KPC and BxPC-3 cell lines were cultured in 1640 medium.

### Subcutaneous tumorigenesis in C57BL/6 mice

Maintaining a suitable cell state is crucial for conducting tumor formation experiments. It is recommended to collect cells during the logarithmic growth phase when the cell density is around 80–90%. To achieve this, the following steps can be taken: [1] The night before cell collection, replace the culture medium with fresh medium. [2] Prior to cell collection, trypsinize the cells and wash them twice with pre-cooled PBS to eliminate serum from the cells. [3] With PBS or serum-free medium to blow the cell precipitation to the appropriate concentration, the general amount of subcutaneous tumor cells inoculated is 1–5 × 10^^^6 cells/mouse, the inoculated volume of 0.1 ml, so the cell suspension concentration of 1–5 × 10^^^7 cells/ml. [4] The cells should be inoculated as soon as possible after digestion in subcutaneous nude mice, generally trying to complete within half an hour, the way the cell suspension on ice reduces cell metabolism. [5] The choice of C57BL/6 mice is generally 5–8 weeks of age, planting site selection of blood-rich areas, such as the middle and rear armpits, and upper groin. [6] Before inoculation, the cell suspension was fully blown away with a gun to prevent cell aggregation and reduce cell survival rate. The maximum diameter of mouse tumors in all animal experiments is less than 15 mm, not exceeding the limits specified in the NIH Guidelines for Endpoints in Animal Study Proposals.

### The Flow cytometry analysis of harvested tumors

The object of flow cytometry is single cell suspension, so the sample should be prepared into cell suspension, cell concentration of 10^5^–10^7^/mL. The prepared single cell suspension can be detected by fluorescence or immunofluorescence labeling. The fundamental principles for sample preparation are as follows: [1] Ensure that fresh liquid and suspension cell samples are promptly prepared and detected. [2] Utilize appropriate methods such as washing, enzyme digestion, or EDTA treatment to eliminate impurities from various cell samples, resulting in the separation of adhered cells to obtain a single cell state. [3] For fresh solid tumor tissue, enzymatic, mechanical, or chemical dispersion can be employed to acquire a sufficient number of single cell suspensions. [4] The single cell suspension should contain no less than 107 cells/mL.

### Gene set enrichment analysis (GSEA)

We performed Gene Set Enrichment Analysis (GSEA) between the high- and low-risk groups to investigate potential molecular mechanisms. The reference gene set selected from the Molecular Signature Database was h.all.v7.2.symbols.gmt, which comprises annotated gene sets known as Hallmarks.

### Quantitative real-time PCR (RT-qPCR)

Total RNA was extracted from the cells using TRIZOL reagent (Macherey–Nagel, Germany), followed by reverse transcription to cDNA using the PrimeScript™ RT reagent kit, as per the manufacturer's instructions. PCR amplification was carried out using the Premix Ex Taq™ kit (Takara), with cycling conditions of 95 °C for 30s, followed by 34 cycles of 95 °C for 5s and 60 ℃ for 30s. The primers and probes were chemically synthesized by Sangon Biotech (China) and are provided in the list below: GLUT1: F 5-GCCATGGAGCCCAGCAGCAA-3; R 5-CGGGGACTCTCGGGGCAGAA-3; GLUT4: F 5-GCCTGTGGCCACTGCTCCTG-3; R 5-GGGGTCTCTGGGCCGGGTAG-3; LDHA: F 5-CCAGTGTGCCTGTATGGAGTG-3; R 5-GCACTCTCAACCACCTGCTTG-3; MCT1:F5-CGCGCCGCAGCTTCTTTCTGTAACATTCAAGAGATGTTACAGAAAGAAGCTGCTTTTTTTTAAT-3;R5-TAAAAAAAAGCAGCTTCTTTCTGTAACATCTCTTGAATGTTACAGAAAGAAGCTGCGG-3; MCT4:F5-CGCGCCGGGATTGGCTACAGCGACATTCAAGAGATGTCGCTGTAGCCAATCCCTTTTTTTTAAT-3;R5-TAAAAAAAAGGGATTGGCTACAGCGACATCTCTTGAATGTCGCTGTAGCCAATCCCG G-3.

### Detection of extracellular acidification rate (ECAR)

Extracellular acidification rate Assay Kit (ECAR Assay Kit) is a kit that uses the synthesized acidification detection fluorescent probe BBcellProbe ® P61 to detect changes in extracellular acidification. The dynamic real-time determination of extracellular acidification can be carried out directly and conveniently by simple mixing based on a fluorescence microplate reader. Real-time measurement of cell glycolysis activity is a reliable method for evaluating cell respiration for metabolic characterization and evaluating the toxic effects of treatment on cell function in a high-throughput form. It can be used for simple kinetic determination on standard microplates using a fluorescence microplate reader. As glycolysis proceeds, pyruvate is converted to lactic acid, which causes the extracellular pH to decrease. The P61 probe can sensitively detect the decrease of pH and increase the fluorescence signal of P61 probe, thus realizing the determination of ECAR. Additionally, Glycolysis Assay [Extracellular Acidification] kit (ab197244) is purchased from https://www.abcam.com/(Abcam company).

### Detection of OCR(oxygen consumption rate)

Pyruvate produced by pyruvate glycolysis undergoes a lactate dehydrogenase reaction to produce lactate, allowing cells to quickly produce ATP without consuming oxygen to meet energy needs. The measurement of hydrogen ions in lactate can indicate changes in anaerobic metabolism.

### Statistical analysis

**Fig.1:**
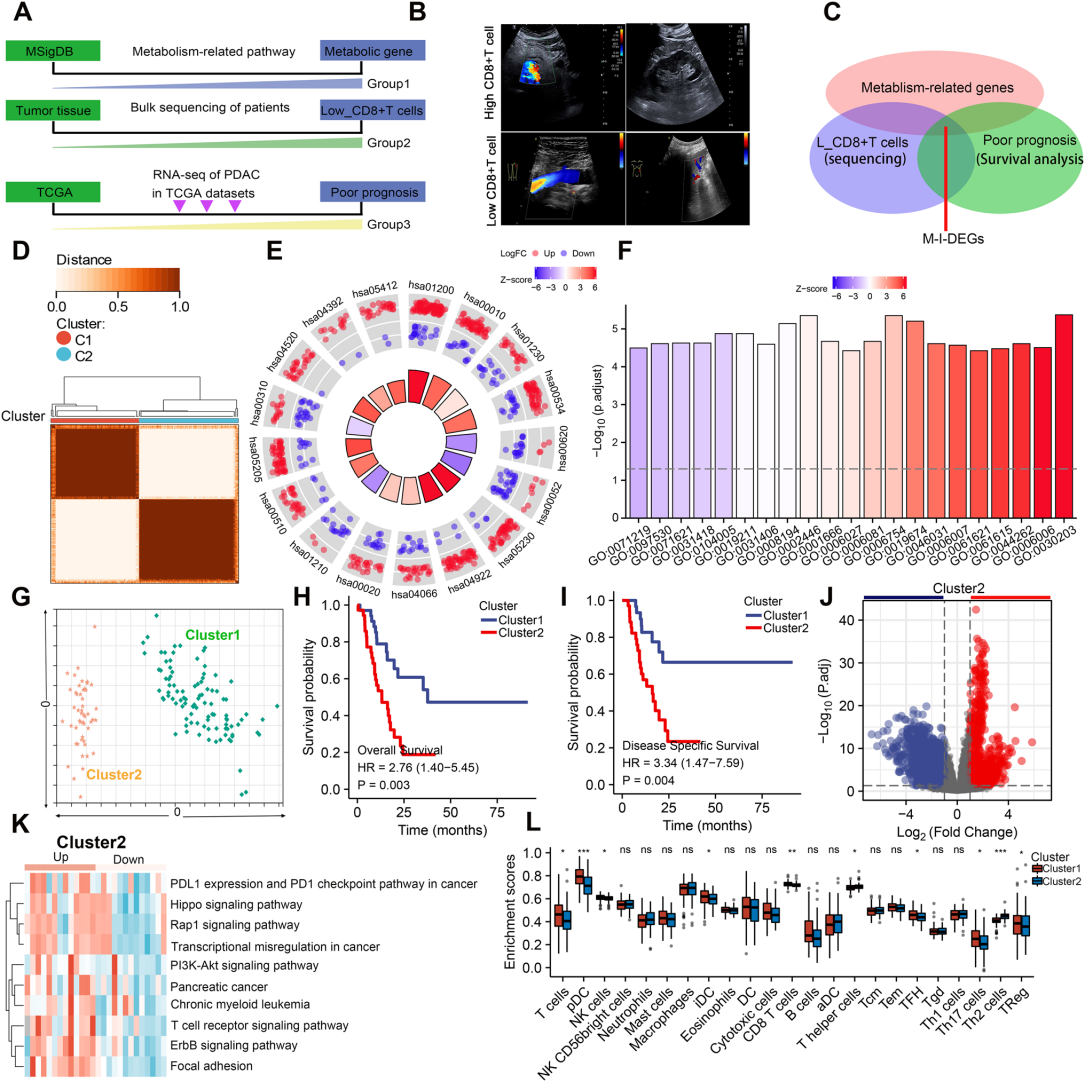
Identification and analysis of metabolism-related differentially expressed genes in PDAC. **a** The flowchart of the screening of differentially expressed genes(DEGs); **b** The postoperative PDAC tissues of six patients who were monitored by ultrasonography were performed with RNA bulk sequencing, and then the analysis of the differentially expressed gene was performed according to the stained intensity of CD8 + T cells in corresponding patients; **c** The intersection of DEGs was shown with venn diagram; all differentially expressed genes were selected according to p.adj value(p.adj < 0.05) and LogFC(LogFC > 1.5); **d** Non-negative Matrix Factorization (NMF) method was used among all samples according to the expression of mDEGs; **e** KEGG analysis was conducted with the DEGs; **f** GO analysis was conducted with the DEGs; **g** Principal component analysis (PCA) of different clusters was conducted; **h**, **i** K-M survival analysis of disease-free survival (DFS; no recurrence/progression) and overall survival (OS) between the two metabolic subtypes was performed; **j** the cluster2 was selected. The up-regulated and down-regulated genes were presented with volcano plot; red colored indicated up-regulated genes and blue colored indicated down-regulated genes; **k** GSVA enrichment analysis was performed between two clusters; red represented activated pathways and blue represented inhibited pathways; **l** The abundance of tumor-infiltrating immune cells was analyzed with CIBERSORT software. We conducted a proportional hazards assumption test and fitted a survival regression using the survival package. The results were visualized using the survminer and ggplot2 packages. If the best grouping method was chosen, the surv_cutpoint function in the survminer package was used to select the optimal cut-off point. Paired student’s t test were performed for in vitro assays and unpaired student’s t test were conducted for in vivo assays; *p < 0.05; **p < 0.01; ***p < 0.001; ****p < 0.0001. The adjusted p-values in Fig. 1e, f refer to the new p-values obtained after multiple comparison correction was applied to the original p-values. In this case, clusterProfiler, an R package, automatically used the false discovery rate (FDR) method to correct the p-values and identify significant pathways for KEGG and GO analyses

Statistical analyses and data visualization in this study were performed using R (version 3.6.3) or GraphPad Prism (version 8.3.0). Continuous variables were compared using t-test, while Fisher’s exact test or chi-square test was used for comparisons of categorical variables. Differences among K-M survival curves were estimated using log-rank test. P-values less than 0.05 (two-tailed) were considered statistically significant. We conducted a proportional hazards assumption test and fitted a survival regression using the survival package. The results were visualized using the survminer and ggplot2 packages. If the best grouping method was chosen, the surv_cutpoint function in the survminer package was used to select the optimal cut-off point. Paired student’s t test were performed for in vitro assays and unpaired student’s t test were conducted for in vivo assays. n.s., no significant statistical difference; *p < 0.05; **p < 0.01; ***p < 0.001; ****p < 0.0001. The adjusted p-values in Figs. 1e, f, 4a, b, and Supplementary Fig. 1b, c refer to the new p-values obtained after multiple comparison correction was applied to the original p-values. In these cases, the false discovery rate (FDR) method was used for p-value correction and to identify significant pathways for visualization. On the other hand, Figs. 3d and 5e–h utilized the Benjamini–Hochberg method for multiple p-value correction.

## Results

### Identification and analysis of metabolism-related differentially expressed genes in PDAC

The metabolic characteristics and microenvironment of pancreatic cancer are also recognized as important factors affecting the efficacy of immunotherapy. The metabolic characteristics of pancreatic cancer primarily include high reliance on glucose metabolism, severe hypoxia and acidosis, lipid metabolism disorders, and abnormal amino acid metabolism. Therefore, screening and predicting markers of pancreatic cancer metabolic characteristics and metabolic microenvironment are beneficial in guiding pancreatic cancer immunotherapy, which can help develop more effective immunotherapy strategies, such as the combination of metabolic intervention and immunotherapy, to enhance the efficacy of immunotherapy [20, 21]. To identify potential intervention targets, we developed a workflow diagram (Fig. 1a) based on the metabolic characteristics and immune microenvironment of pancreatic cancer to screen genes that are significantly positively correlated with both tumor metabolism and immunosuppressive phenotypes. We first collected 1385 metabolism-related genes from the MSigDB database and then selected six PDAC patients who underwent surgery and were monitored for postoperative recurrence by ultrasound. The tissue of these six postoperative PDAC patients was subjected to RNA bulk sequencing (Supplementary table 1), and their immune status was evaluated by CD8 staining on pathological sections (Fig. 1b). Finally, we selected differentially expressed genes based on the staining intensity of CD8 + T cells in corresponding patients and obtained metabolism-related genes that were closely associated with low CD8 + T cell infiltration. In addition, we extracted prognostic-related genes from 178 PDAC patients in the TCGA database and obtained a metabolic-immune-related gene set (M-I-DEGs) by intersecting the three gene sets (Fig. 1c; Supplementary table 2). Next, we used the non-negative matrix factorization (NMF) method to group the expression of the M-I-DEGs and obtained two clusters of patients with functional differences (cluster1, M-I-DEGs_L; cluster2, M-I-DEGs_H) (Fig. 1d). Subsequently, we performed KEGG enrichment analysis on the different gene sets of cluster 1 and cluster 2. The enrichment analysis results showed that the high expression of M-I-DEGs in cluster 2 was significantly associated with pathways such as carbon metabolism, glycerophospholipid metabolism, amino acid biosynthesis, and cerebellar ataxia (Fig. 1e). The top five GO enrichment analysis results for cluster2 patients were fatty acid metabolism, small molecule organic compound catabolic process, glycerolipid metabolism, phospholipid metabolism, and organic acid biosynthetic process (Fig. 1f). We further performed principal component analysis (PCA) on the M-I-DEGs of the two clustering clusters, which showed good independence between them (Fig. 1g). To investigate the biological behavioral differences between the two clustering clusters, we performed survival analysis between the two groups and found that the high expression of M-I-DEGs in cluster2 was significantly associated with shorter overall survival (OS) and disease-free survival (DFS) (Fig. 1h, i). At the same time, we obtained the DEGs that were highly expressed in the cluster2 samples and presented them using a volcano plot (Fig. 1j). We further explored the functional enrichment of upregulated and downregulated genes in cluster2 using Gene Set Variation Analysis (GSVA) and visualized the top 13 significant biological processes (Fig. 1k). Our results showed that the upregulated genes in cluster2 mainly participated in pathways such as PDL1 expression and PD1 checkpoint pathway in cancer, PI3K-Akt signaling pathway, Hippo signaling pathway, and pancreatic cancer. Finally, we analyzed the abundance of tumor-infiltrating immune cells in the two clusters and found that activated B cells, NK cells, dendritic cells, activated CD4 + T cells, and activated CD8 + T cells were significantly decreased in the cluster2 group (Fig. 1l). Our research results demonstrate that the screened M-I-DEGs can affect the metabolism and immune microenvironment of pancreatic cancer and predict the prognosis of pancreatic cancer. We will continue to search for a more meaningful target that can predict the metabolic and immune microenvironment characteristics of pancreatic cancer and guide the immune checkpoint inhibition therapy for pancreatic cancer. In the future, metabolic interventions based on this target may improve the effectiveness of PDAC immunotherapy and further enrich the feasibility of pancreatic cancer immunotherapy.

### Construction of a metabolism-related risk model to predict the disease-free survival of pdac patients

**Fig. 2:**
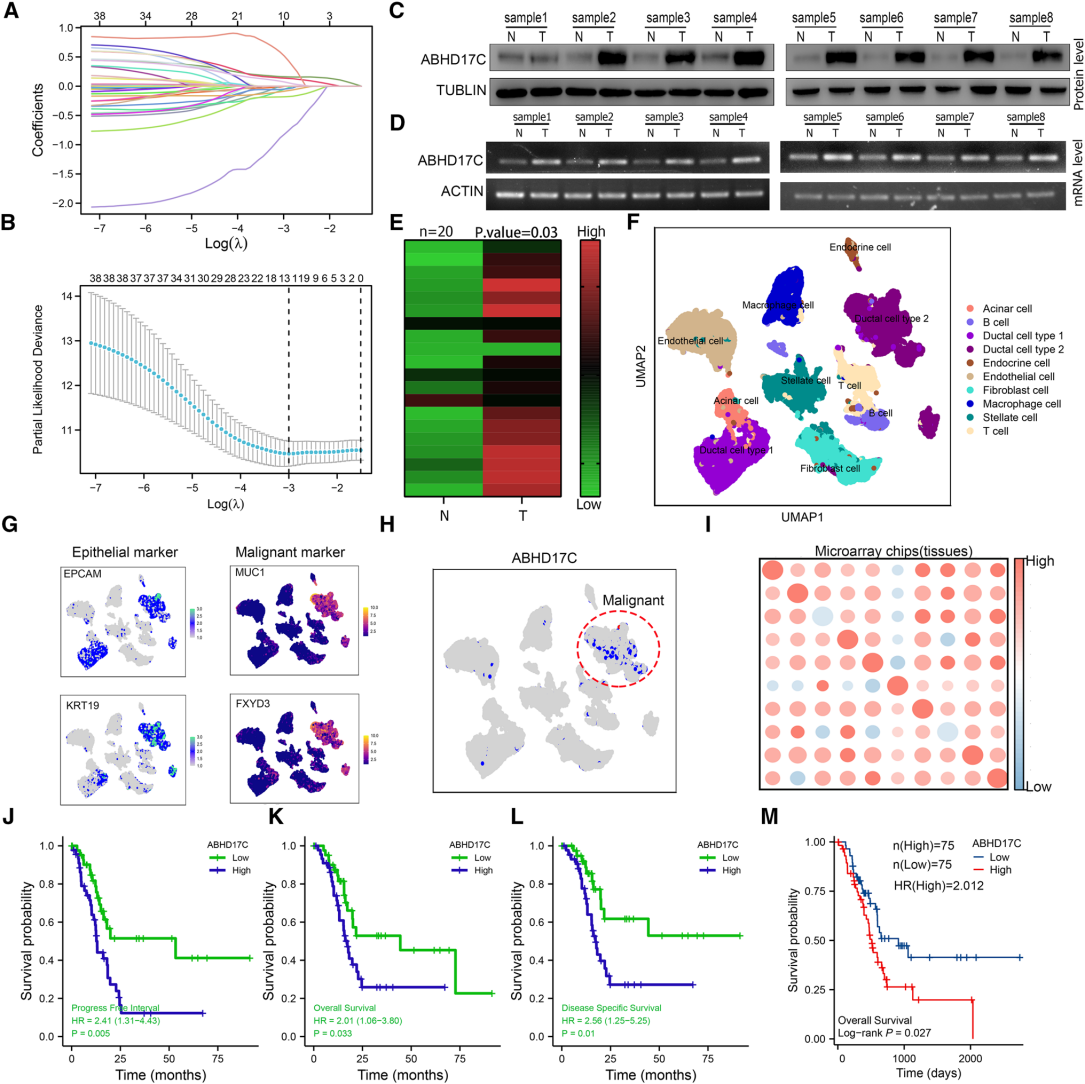
Construction of a Metabolism-Related Risk Model to Predict the Disease-Free Survival of PDAC Patients. **a**, **b** The Lasso-Cox regression analysis was performed with 48 intersecting genes in cluster 2; the minimum partial likelihood deviance was selected to identify the targeted marker, ABHD17C. **c**, **d** Western blot and PCR assays in eight paired tumor tissues and adjacent normal pancreatic tissues were performed to verify the expression of ABHD17C; **e** IHC analysis in paired tumor tissues and adjacent normal pancreatic tissues was conducted to estimate the expression intensity of ABHD17C; the results were shown with heatmap. **f** The single cell RNA sequencing datasets downloaded from CRA10016 were analyzed with Seurat software, and displayed with UMAP plot; all cell types were color coded. **g** The malignant ductal cells were identified with KRT19, EPCAM, FXYD3, and MUC1 markers; all selected markers were presented with UMAP plot. **h** ABHD17C was color-coded and presented with UMAP plot. **i** we performed IHC analysis in the microarray chip which contains one hundred tumor tissue samples; The one hundred samples were divided into two groups according to the expression of ABHD17C. **j**–**l** The K-M analysis of our data was conducted in patients with high ABHD17C expression compared with patients with low ABHD17C expression; the overall survival (OS) time, disease-free survival (DFS) time, and progression-free interval (PFI) time were calculated in two groups. **m** The K–M analysis of public data in the TCGA database was conducted in patients with high ABHD17C expression compared with patients with low ABHD17C expression; the overall survival (OS) time and disease-free survival (DFS) time were calculated. We conducted a proportional hazards assumption test and fitted a survival regression using the survival package. The results were visualized using the survminer and ggplot2 packages. If the best grouping method was chosen, the surv_cutpoint function in the survminer package was used to select the optimal cut-off point. Paired student’s t test were performed for in vitro assays and unpaired student’s t test were conducted for in vivo assays. *p < 0.05; **p < 0.01; ***p < 0.001; ****p < 0.0001

To identify a comprehensive and effective metabolic risk marker, we continued to select 48 cross-genes and then performed LASSO-Cox regression analysis on key metabolic differential genes related to overall survival. After cross-validation, ABHD17C was identified as the target gene with the minimum deviation probability that influences pancreatic cancer survival prognosis (Fig. 2a, b). First, we prospectively selected eight paired pancreatic ductal adenocarcinoma (PDAC) and adjacent tissue samples for validation of ABHD17C expression levels. Our results showed that ABHD17C was significantly increased at the protein level in eight prospective PDAC tissues compared with adjacent normal tissues, as well as at the mRNA level, which was evaluated by gel electrophoresis after PCR product quantification (Fig. 2c-d). Subsequently, we retrospectively performed immunohistochemical analysis on 20 pairs of PDAC tissues and adjacent normal pancreatic tissues. The researchers found that ABHD17C was more upregulated in tumor tissues relative to adjacent normal tissues (Fig. 2e). To further increase the strength of the evidence, we downloaded single cell transcriptome sequencing data for 24 cases of pancreatic ductal adenocarcinoma from PRJCA003818. We selected KRT19 and EPCAM as markers for all ductal epithelial cells and MUC1 and FXYD3 as markers for malignant ducts to distinguish between malignant and benign ducts. Single cell transcriptome sequencing analysis showed that ABHD17C expression was significantly upregulated in malignant ductal cells (Fig. 2f–h). However, the biological malignant behavior and risk value of ABHD17C in PDAC patients at our center are still unclear. We further performed IHC analysis on PDAC samples from 100 tumor tissue samples on a microarray chip at our center. Based on the expression level of ABHD17C, tumor tissues were divided into ABHD17C low-expression and ABHD17C high-expression groups. The correlation analysis showed that ABHD17C high expression was positively correlated with tumor size (R = 0.520, p = 0.000), histological grade (R = 0.221, p = 0.031), TNM stage (R = 0.205, p = 0.034), and histological grade of tumor tissue (R = 0.226, p = 0.031) in PDAC patients (Supplementary table 3). The K-M analysis showed that patients with high expression of ABHD17C had significantly lower rates of OS, DSS, and PFI than those with low expression of ABHD17C (Fig. 2j, l). Furthermore, we performed additional K-M analysis in 150 PDAC patients from the public TCGA database, which provided similar results (Fig. 2m; Supplementary Fig. 3a, b; Supplementary table 4, 5). Taken together, the results from our center and public databases indicated that the expression of ABHD17C was negatively correlated with OS, DSS, and PFI in PDAC patients, and it could serve as a valuable prognostic marker for predicting PDAC development and progression. To further validate the function of ABHD17C, we conducted KEGG and GO pathway enrichment analysis and immune infiltration analysis using the previously downloaded single cell transcriptome sequencing data. Our results showed that high expression of ABHD17C in malignant ductal cells was positively correlated with metabolic processes and could be enriched in cancer-related signaling pathways such as MAPK signaling pathway, protein glycosylation, Wnt signaling pathway, and PI3K-Akt signaling pathway (Supplementary Fig. 1a–c). In addition, our results indicated that ABHD17C from tumor cells could reduce the infiltration of cytotoxic T cells and increase the levels of Tregs, TAMs, and granulocytic cells, which may exacerbate the immune-suppressive microenvironment and promote tumor burden (Supplementary Fig. 2a).

### Overexpression of ABHD17C remodels the information of the immune suppressive environment

**Fig. 3:**
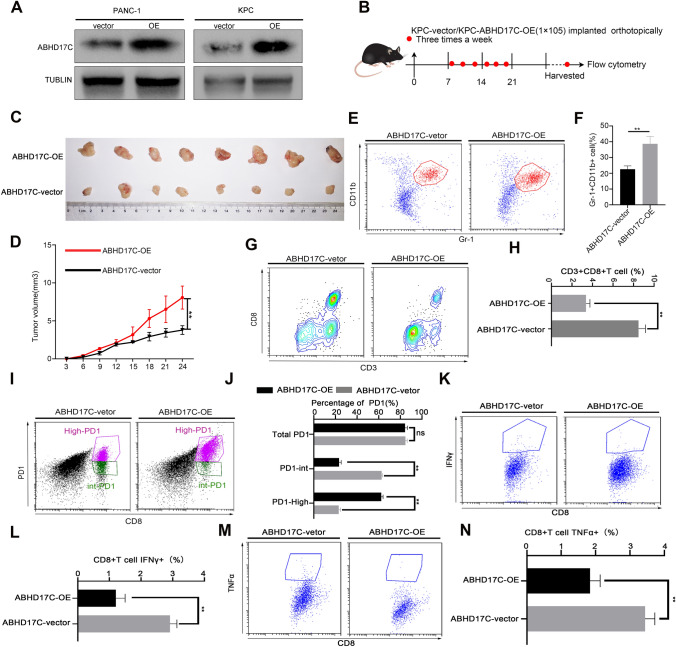
Overexpression of ABHD17C remodels the information of the immune suppressive environment. **a** the KPC murine cell lines and PANC1 human cell lines which stably expressed ABHD17C were constructed; the validation of ABHD17C expression was presented with western blot; **b** the murine KPC-ABHD17C-vector/OE cell lines were constructed and injected subcutaneously into C57/BL mouse, and the tumor size was measured after implantation three times a week; **c**, **d** The tumor size was monitored and the growth curve was recorded; **e**–**n** the harvested tumors were performed with flow cytometry analysis; Representative dot plots and statistical analysis of the frequency of MDSCs, and CD3 + CD8 + T cells. Representative dot plots and statistical analysis of the frequency of tumor-infiltrating CD8 + IFNγ + T cells, CD8 + PD1 + T cells, and CD8 + Grzmb + T cells. Experiments were repeated three times independently. Representative data are shown. Data are presented as mean ± SD. We conducted a proportional hazards assumption test and fitted a survival regression using the survival package. Paired student’s t test were performed for in vitro assays and unpaired student’s t test were conducted for in vivo assays. n.s., no significant statistical difference; *p < 0.05; **p < 0.01; ***p < 0.001; ****p < 0.0001. In **c**, **d**, the tumor volume was calculated based on the long and short diameters measured at multiple time points for both the control and experimental groups. The Benjamini–Hochberg method was used for multiple p-value correction

Based on the screening strategy and analysis results mentioned above, ABHD17C is a critical prognostic and diagnostic marker for pancreatic cancer and is closely associated with the immunosuppressive microenvironment and metabolic state of pancreatic cancer. To further determine the function of ABHD17C in reshaping the immunosuppressive environment of pancreatic cancer in vitro and in vivo, we first constructed stable mouse-derived KPC-ABHD17C-Vector/OE cell lines and human-derived PANC-1-ABHD17C-Vector/OE cell lines (Fig. 3a). Subsequently, the KPC-ABHD17C-Vector/OE cell lines were subcutaneously injected into immunocompetent C57/BL mice, and the tumor size was measured three times a week to generate growth curves based on the measured data (Fig. 3b). Our results showed that the upregulation of ABHD17C significantly increased tumor burden (Fig. 3c, d). We also investigated the effect of ABHD17C overexpression on the proportion of different types of immune cells in the tumor microenvironment by analyzing the collected tumors using flow cytometry. Our results revealed that the infiltration of myeloid-derived suppressor cells (MDSCs) was significantly increased in the KPC-ABHD17C-OE group compared to the KPC-ABHD17C-Vector group (Fig. 3e, f). Furthermore, our additional results showed that the percentage of CD8 + T cells and the percentage of cytotoxic factors (TNFα and IFNγ) were significantly decreased in the KPC-ABHD17C-OE group compared to the KPC-ABHD17C-Vector group (Fig. 3g–h, k–n), whereas the percentage of CD8 + PD1 + T cells was significantly increased (Fig. 3i, j). Based on these results, we concluded that MDSCs are bone marrow-derived cells that typically exert immunosuppressive functions, and an increase in the proportion of MDSCs due to ABHD17C overexpression leads to the formation of an immunosuppressive microenvironment in pancreatic cancer. CD8 + T cells, on the other hand, are effector cells that exert specific killing effects, and the secretion of TNFα and IFNγ can mediate the killing function of CD8 + T cells. However, high PD1 expression leads to CD8 + T cell exhaustion, and an increase in PD1 + CD8 + T cells and a decrease in TNFα + /IFNγ + CD8 + T cells due to ABHD17C in the pancreatic cancer microenvironment can promote the immune escape of tumor cells and inhibit immune killing. Therefore, based on the above research results, the elevation of ABHD17C in pancreatic cancer cells may promote the formation of an immunosuppressive environment, increase tumor burden, and promote the occurrence and development of pancreatic cancer.

### ABHD17C was positively correlated with the glycolytic process of tumor cells in PDAC

**Fig. 4:**
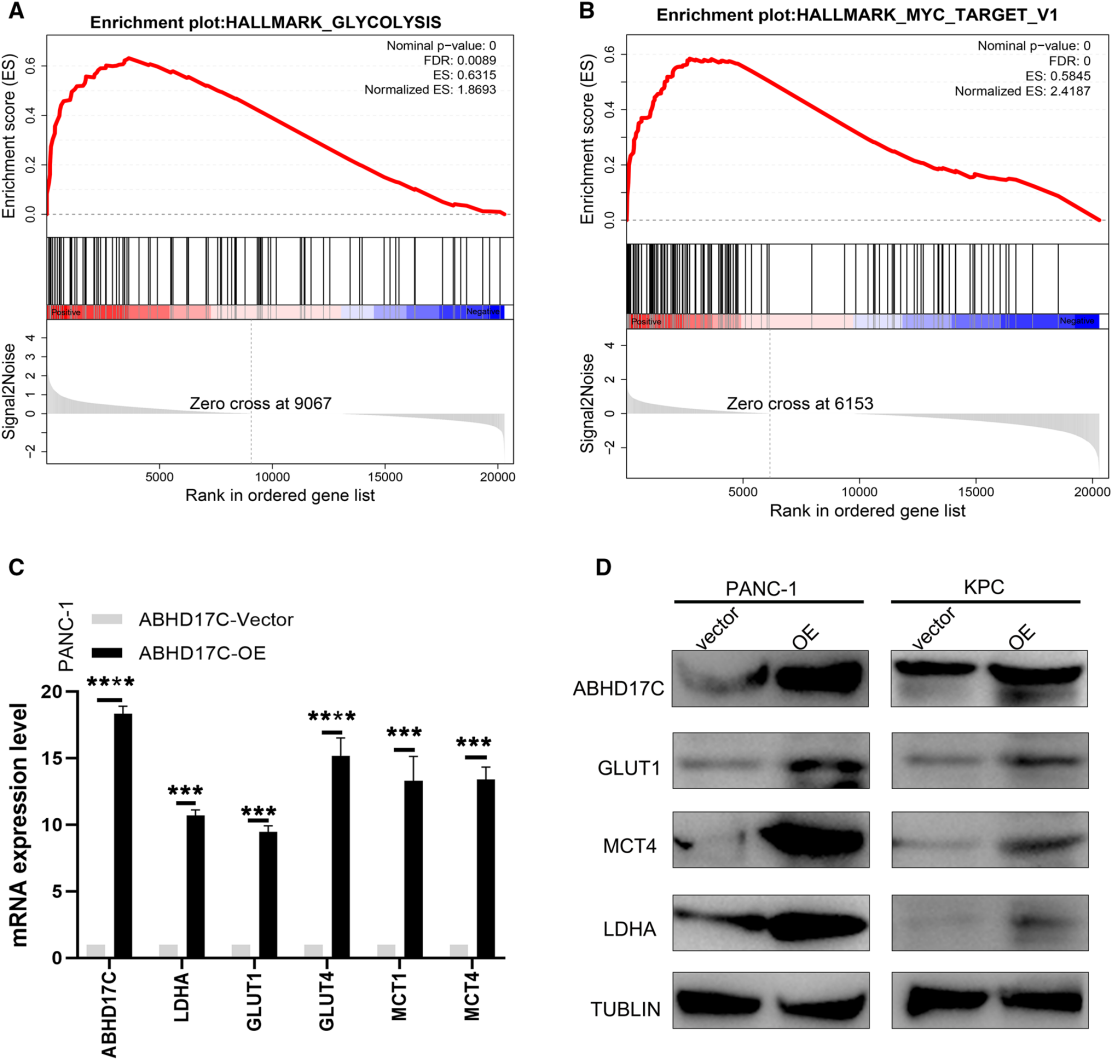
ABHD17C was positively correlated with the glycolytic process of tumor cells in PDAC. **a**, **b** Gene set enrichment analysis (GSEA) was conducted based on the mRNA expression level of ABHD17C in ten PDAC tissues in our center. We then filtered significantly differentially enriched pathways based on the adj. p-value and enrichment score(ES), and the adjusted p-value < 0.05 was considered to represent a significant difference; **c** QPCR assay was conducted to estimate the relationship between ABHD17C and metabolic markers(MCT1, MCT4, LDHA, GLUT1, and GLUT4). Experiments were repeated three times independently. Representative data are shown. Data are presented as mean ± SD. **P < 0.01; ***P < 0.001; ****P < 0.0001; n. s., no significant statistical difference. **d** Western blot assay was performed to identify the relative expression of metabolic markers (MCT1, MCT4, LDHA, GLUT1, and GLUT4) between ABHD17C-vector and ABHD17C-OE group. Paired student’s t test were performed for in vitro assays and unpaired student’s t test were conducted for in vivo assays. n.s., no significant statistical difference; *p < 0.05; **p < 0.01; ***p < 0.001; ****p < 0.0001. In **a**, **b**, the adjusted p-values refer to the new p-values obtained after multiple comparison correction was applied to the original p-values in GSEA analysis. The R package GSEA automatically used the FDR method to correct the p-values and identify significant pathways

According to the results presented in Fig. 3, the increase of tumor-derived ABHD17C may promote the development of pancreatic cancer by reshaping the immunosuppressive microenvironment. However, it is unclear how ABHD17C, a tumor-derived factor, influences the pancreatic cancer microenvironment. To further understand the potential mechanisms underlying the increased tumor burden and accelerated formation of the immunosuppressive microenvironment by ABHD17C, we performed GSEA enrichment analysis on the mRNA sequencing results of PANC-1-ABHD17C-Vector and PANC-1-ABHD17C-OE cell lines. Our enrichment analysis revealed that overexpression of ABHD17C significantly enriched the HALLMARK_GLYCOLYSIS pathway (NES = 1.8693, Nominal P-value = 0, FDR = 0.0089) and the HALLMARK_MYC_TARGET_V1 pathway (NES = 2.4187, Nominal P-value = 0, FDR = 0.0). The HALLMARK_MYC and HALLMARK_GLYCOLYSIS pathways are critical metabolic pathways in glycolysis, indicating that the overexpression of ABHD17C is positively correlated with the glycolysis process of pancreatic cancer tumor cells (Fig. 4a, b).

To validate our conclusion, we selected key effectors in the two enriched pathways, MCT1, MCT4, GLUT1, GLUT4, and LDHA, for verification. Our qPCR and western blotting results showed that the elevation of ABHD17C could increase the expression of MCT1, MCT4, GLUT1, GLUT4, and LDHA at both the protein and mRNA levels (Fig. 4c, d). MCT1 and MCT4 are classic lactate transport proteins, GLUT1 and GLUT4 are key glucose uptake channel proteins, and LDHA is a key molecule involved in the later stage of lactate metabolism in glycolysis. In summary, our results demonstrate that ABHD17C is closely related to the glycolysis process, which may be a potential mechanism underlying its effects on the pancreatic cancer immune microenvironment. However, further functional experiments on glycolysis are needed to support this conclusion.

### Elevation of ABHD17C increases the glycolytic ability of tumor cells in PDAC

**Fig.5:**
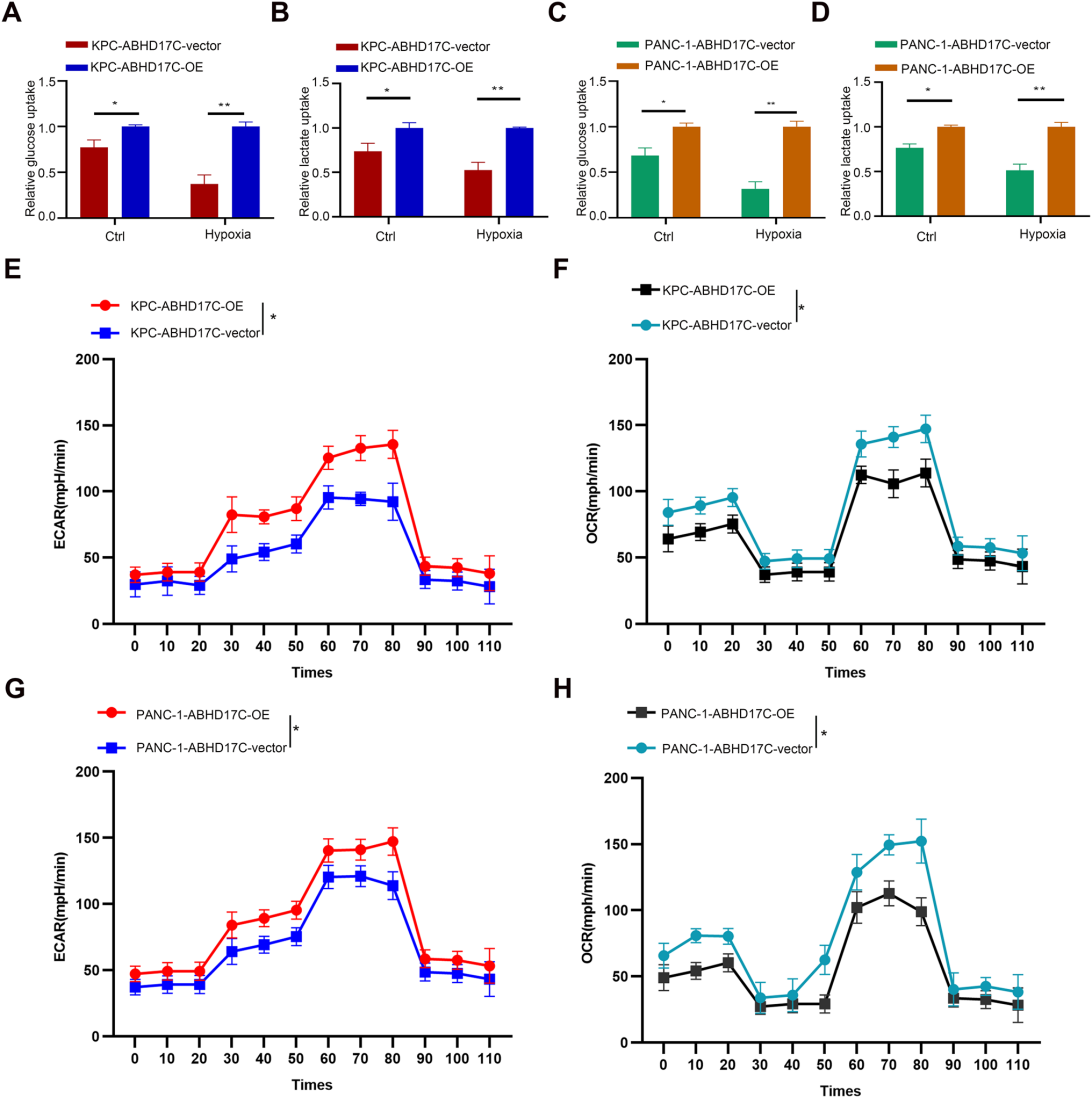
Elevation of ABHD17C increases the glycolytic ability of tumor cells in PDAC. **a**–**d** the relative ratio of lactate excretion and glucose uptake was calculated in indicated cell lines. Experiments were repeated three times independently. Representative data are shown. Data are presented as mean ± SD. **e**–**h** ECAR and OCR assays which represented the potential ability of glycolysis were performed between PANC-1(KPC)-ABHD17C-vector and PANC(KPC)-1-ABHD17C-OE cell lines; Paired student’s t test were performed for in vitro assays and unpaired student’s t test were conducted for in vivo assays. n.s., no significant statistical difference; *p < 0.05; **p < 0.01; ***p < 0.001; ****p < 0.0001. In Fig. 5e–h, the maximum glycolytic capacity and basal energy storage were measured at multiple time points for both the control and experimental groups and the Benjamini–Hochberg method was used for multiple p-value correction

In Fig. 4, we observed a significant positive correlation between tumor-derived ABHD17C and glycolysis. However, more convincing evidence is needed to directly demonstrate that ABHD17C promotes glucose metabolism in pancreatic cancer cells. Therefore, we conducted four classical glycolysis-related functional experiments to provide further evidence: glucose uptake assay, lactate secretion assay, OCR, and ECAR experiments. Previous studies have shown that increased glycolytic capacity in tumor cells leads to increased glucose uptake and lactate excretion, while ECAR and OCR are key experiments that reflect glycolytic capacity. The results of glucose uptake and lactate metabolism assays indicated that the elevation of tumor-derived ABHD17C significantly increased glucose uptake and lactate secretion (Fig. 5a–d). Subsequently, ECAR and OCR experiments, which represent the potential storage capacity and maximum storage capacity of glycolysis, demonstrated that the overexpression of ABHD17C increased the glycolysis level of pancreatic cancer cells (Fig. 5e–h). These glycolysis-related functional experiments indicate that ABHD17C promotes glycolysis levels by increasing the glycolytic storage capacity of pancreatic cancer cells, thereby enhancing the glucose uptake ability and lactate secretion level of tumor cells, leading to a significant decrease in the pH value of the microenvironment. This may be a key factor in the reshaping of the immune microenvironment. Next, we need to conduct blocking experiments to further confirm our results.

### The aggravation of tumor burden caused by ABHD17C was inhibited by LDHA inhibition which resulted in glycolysis defect

**Fig.6:**
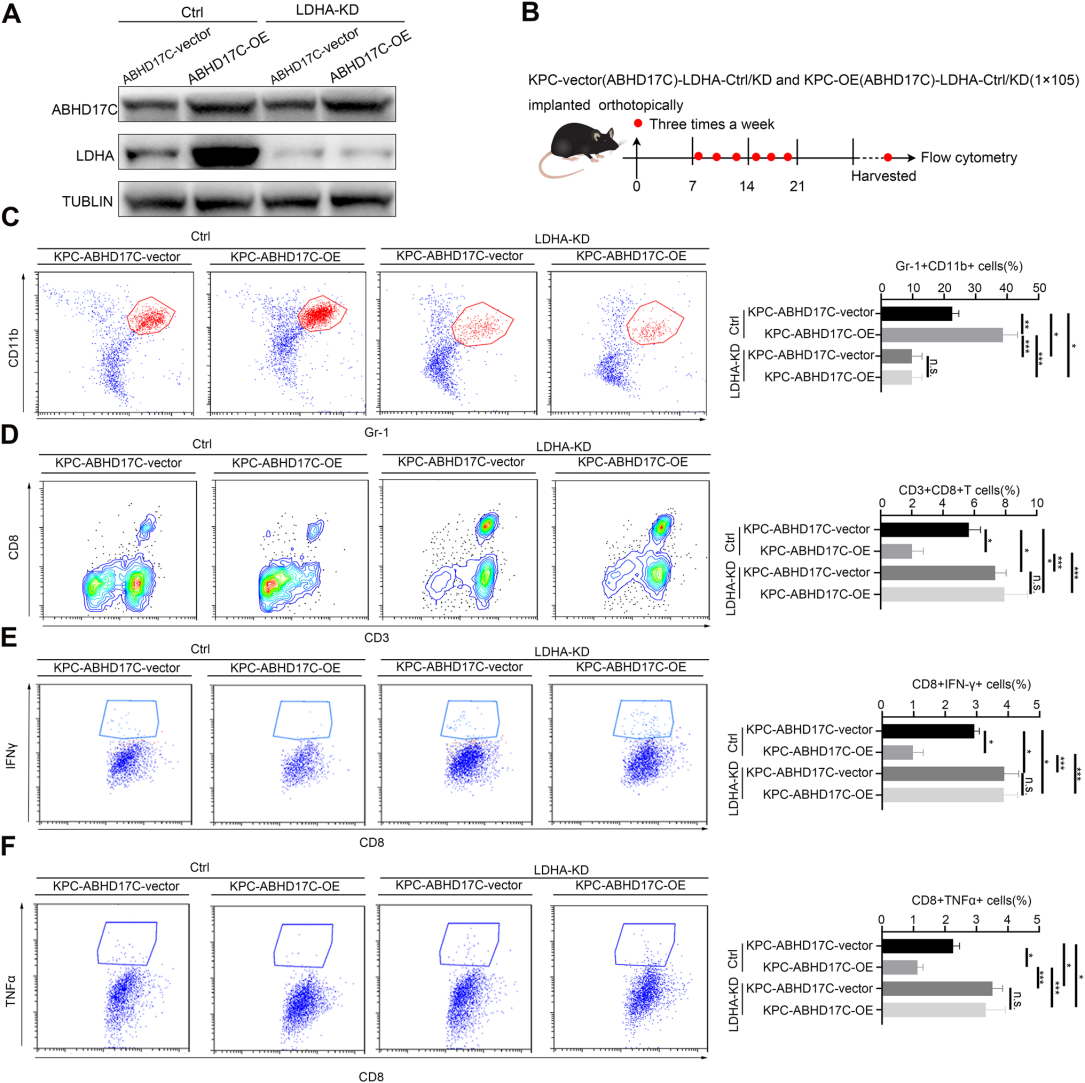
The aggravation of tumor burden caused by ABHD17C was inhibited by LDHA inhibition which resulted in glycolysis defect. **a** the murine KPC-ABHD17C-vector/OE-LDHA-vector/KD cell lines were constructed and validated with western blot assay; **b** the murine KPC-ABHD17C-vector/OE-LDHA-vector/KD cell lines were injected subcutaneously into C57/BL mouse; the subcutaneous tumors were randomly divided into KPC-ABHD17C-vector/OE -vector group and KPC-ABHD17C-vector/OE-LDHA-KD group; **c**–**f** the harvested tumors were performed with flow cytometry analysis; Representative dot plots and statistical analysis of the frequency of tumor-infiltrating T reg cells, MDSCs, and CD3 + CD8 + T cells. Representative dot plots and statistical analysis of the frequency of tumor-infiltrating CD8 + IFNγ + T cells, CD8 + PD1 + T cells, and CD8 + Grzmb + T cells. Experiments were repeated three times independently. Representative data are shown*. *Data are presented as mean ± SD. Paired student’s t test were performed for in vitro assays and unpaired student’s t test were conducted for in vivo assays. n.s., no significant statistical difference; *p < 0.05; **p < 0.01; ***p < 0.001; ****p < 0.0001

However, the mechanism of the alleviation of tumor burden caused by ABHD17C was not clearly figured out in this research. Subsequently, the key glycolytic gene, LDHA, was identified as a key enzyme subunit for lactic acid production, and the knockout of LDHA could result in glycolysis defects. So KPC-ABHD17C-vector-LDHA-Ctrl, KPC-ABHD17C-vector-LDHA-KD, KPC-ABHD17C-OE-LDHA-Ctrl, KPC-ABHD17C-OE-LDHA-KD were constructed for subsequent research (Fig. 6a), and the growth of KPC-ABHD17C-OE-LDHA-KD tumor was significantly slower than that of KPC-ABHD17C-OE-LDHA-Ctrl with complete glycolysis (Fig. 6b, Supplementary Fig. 4a, b). Flow cytometry analysis of harvested tumor indicated that the analysis observed that the infiltration of MDSCs significantly reduced in KPC-ABHD17C-OE-LDHA-KD group than that in KPC-ABHD17C-vector-LDHA-Ctrl group (Fig. 6c). Besides the proportion of CD8 + T cells level and the percentage of functional factors in CD8 + T cells notably elevated in KPC-LDHA-KD group compared with that in KPC-LDHA-Ctrl group (Fig. 6d–f). In summary, we have demonstrated that inhibition of the key gene LDHA in the tumor glycolytic metabolism significantly improves the immune microenvironment and delays tumor burden increase in the context of ABHD17C overexpression. These findings suggest that the immunosuppressive microenvironment formation and tumor burden increase caused by ABHD17C are mainly attributed to the elevation of glycolytic level in pancreatic cancer.

### ABHD17C expression could predict the efficacy of anti-PD1 therapy in pancreatic cancer

**Fig. 7:**
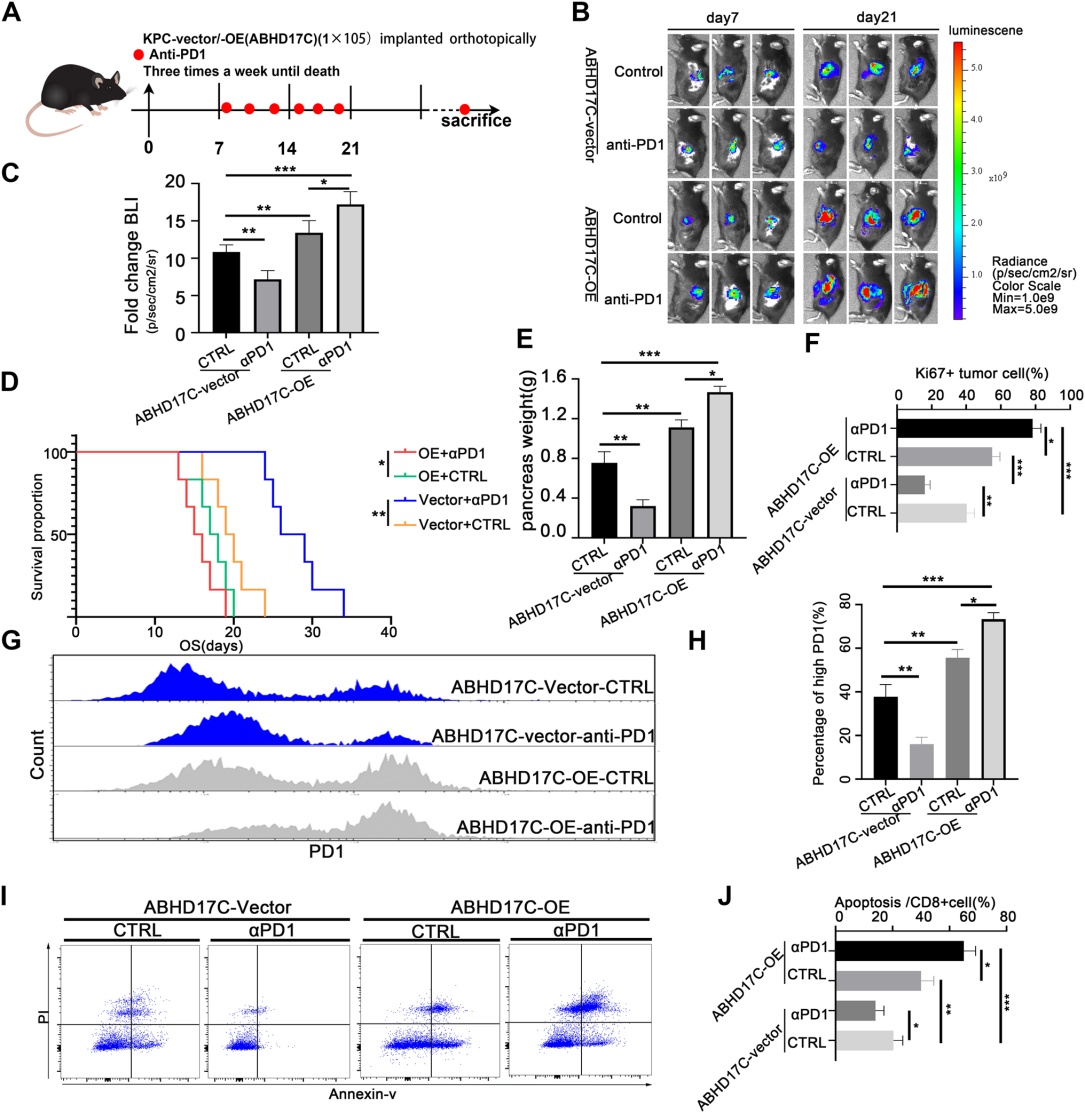
Anti-PD1 therapy inhibited tumor proliferation and improved the immune microenvironment in ABDH17C-deficient pancreatic cancer. **a** KPC-ABDH17C-vector/OE cell lines were implanted orthotopically in C57BL / 6 immunocompetent mice, the blocking antibody of PD1 was used for intraperitoneal injection (red dots represented the beginning of administration); three times a week until the mice sacrificed. **b** Tumors implanted orthotopically were detected in vivo by bioluminescent imaging on day 7 and 21; **c** statistical analysis of the fold change of bioluminescent value was calculated; **d** the K-M survival analysis between KPC-vector and KPC-OE groups was performed until the sacrifice of mice. The color of the Bar represents the range of fluorescence intensity change; **P < 0.01; n. s., no significant statistical difference; the data are presented by mean + -SD. **e** pancreas weight(g) was calculated among four groups, and shown with a bar plot; **f** the percentage of ki67 + tumor cells was calculated; the data were statistically analyzed by histogram; **g**, **h** The infiltration ratio of CD8 + PD1 + T cells was detected by flow cytometry; the Y axis represents CD8 + T cell, and the X axis represents high PD1 + . **i**,** j** The apoptosis ratio of CD8 + T cells was detected by flow cytometry; the Y axis represents PI gated, and the X axis represents APC; the data were statistically analyzed by histogram; the mean + SD was used for statistical analysis between groups, **P < 0.01; *P < 0.05; ***P < 0.001;n. s., no significant statistical difference

In our previous study, we noticed that the elevation of ABHD17C could increase the infiltration of MDSCs, and tumoral ABHD17C could promote the remodel of an immunosuppressive environment. Our results speculated that the expression of ABHD17C in KPC cells could efficiently predict the efficacy of anti-PD1 therapy. To confirm these effects, we performed the correlation between tumoral ABHD17C and the efficacy of anti-PD1 therapy (Fig. 7a, b); We first searched for datasets of pancreatic cancer patients treated with Anti-PD1/CTLA-4/PD-L1. Based on the expression of ABHD17C and the corresponding patients' responsiveness to ICB treatment, we calculated the specificity and sensitivity of predicting ICB therapy for patients with high ABHD17C expression and plotted the ROC curve (Supplementary Fig. 5a, b). Additionally, we plotted survival curves between the high and low ABHD17C expression groups (Supplementary Fig. 5c). The ROC analysis in three cohorts(Riaz cohort 2018, A; Gao cohort 2018, B; Cho cohort 2020, C) and drug sensitivity analysis in public datasets inferred that overexpression of ABHD17C increased the resistance of anti-PD1 therapy (Supplementary Fig. 5). Subsequently, our results indicated the BLI Fluorescence value of anti-PD1 treatment significantly decreased in the KPC—ABHD17C-Vector group, but no significant decrease in the KPC—ABHD17C-OE group in the orthotopic C57BL/6 tumor mice model (Fig. 7c); moreover, anti-PD1 treatment could significantly prolong the survival time in KPC-ABHD17C-Vector group, comparing with KPC-ABHD17C-OE group (Fig. 7d). We inferred that one of the main reasons for inhibiting tumor proliferation was that the deficiency of ABHD17C caused less infiltration of MDSCs and was also accompanied by changes in the immune microenvironment combined with anti-PD1 therapy. To further explore the changes in the immune microenvironment, we detected the infiltration and functional changes of CD8 + T cells among four groups by flow cytometry. The results firstly showed the proportion of Ki67 + tumor cells in Vector group was significantly reduced than that in OE group after anti-PD1 treatment (Fig. 7e, f). Then, we further detected the change in PD1 expression level in CD8 + T cells. The results indicated Vector group had a high PD1 level, which was non-sensitive to anti-PD1 therapy; and the high PD1 level of CD8 + T cells significantly decreased in the Vector group compared with OE group after anti-PD1 treatment (Fig. 7g, h); Moreover, comparing with the ABHD17C-OE group, the apoptosis ratio of CD8 + T cells in the ABHD17C-Vector group also significantly reduced (Fig. 7i, j). In addition, the TUNEL and Ki67 immunofluorescence staining on tumors implanted in mice demonstrated that the overexpression of ABHD17C was strongly associated with the resistance to Anti-PD1 therapy (Supplementary Fig. 5a–d). These findings provide strong evidence that ABHD17C plays a crucial role in modulating the resistance of ICB-based therapy by remodeling the suppressive immune environment in PDAC.

**Fig. 8:**
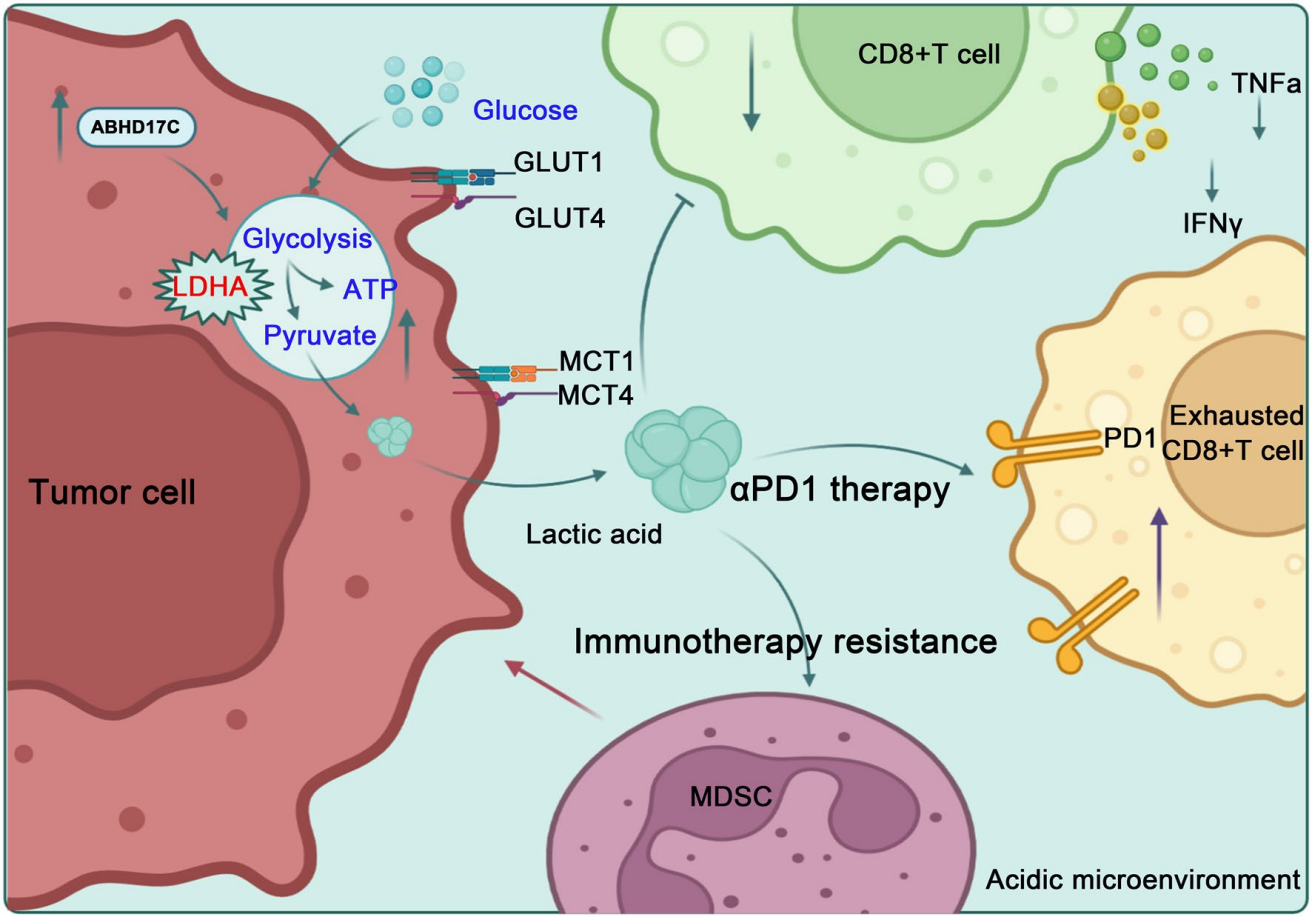
A schematic diagram of this research

As our schematic diagram of this research shows (Fig. 8a), on the one hand, the elevation of tumor-derived ABHD17C in PDAC could significantly increase the expression of MCT1, MCT4, GLUT1, GLUT4, and LDHA at protein and mRNA levels, which increased glycolytic levels of tumor cells and the production of acidic metabolites in the microenvironment. The change of microenvironment caused by ABHD17C could promote the chemotaxis of MDSCs and inhibit the function of CD8 + T cells, resulting in an immunosuppressive microenvironment; on the other hand, the inhibition of the function of CD8 + T cells and increased proportion of MDSCs resulted by ABHD17C could increase the resistance of anti-PD1 therapy. Finally, the deficiency of tumoral-derived ABHD17C could sensitize anti-PD1 therapy and could be expected to be a potential marker for the prediction of anti-PD1 therapy in pancreatic cancer.

## Discussion

The 5-year survival rate of pancreatic cancer is only 8%, and the reason was closely related to the late discovery and early diagnosis difficulties [22]. Pancreatic ductal adenocarcinoma is the most common pathological type of pancreatic cancer. The pathological manifestations are the disorder of gland arrangement, nuclear pleomorphism, incomplete gland cavity, necrosis, gland invasion of blood vessels, neurophilic invasion, and lymphatic invasion [3].

Cell metabolic reprogramming is an important factor in tumorigenesis, which contributes to the occurrence and development of tumors [23]. With the changes in intracellular and extracellular metabolites, metabolic reprogramming has a profound impact on gene expression, cell heterogeneity, and TME [24]. Metabolic reprogramming also occurs in PDAC and exhibits a unique metabolism, although PDAC is different and self-limiting [21]. Therefore, the establishment of effective prognostic labels is crucial for the evaluation and treatment of PDAC.

In this study, we first identified and analyzed mDEGs (metabolism-related DEGs) in the PDAC database, which are enriched in lipid and amino acid-related metabolic processes. Based on these DEGs, consensus clustering analysis found that patients could be divided into two subgroups, and there were significant differences in DFS and OS between the two subgroups. These results indicate that the metabolism of PDAC is uneven, and patients with different metabolic patterns have different outcomes.

Subsequently, a key survival-related m-DEGs (ABHD17C), a new targeted gene, was screened by Least absolute shrinkage and selection operator (lasso) analysis. ABHD17C [25] (Abhydrolase Domain Containing 17C, Depalmitoylase) is a Protein Coding gene. Among its related pathways are RAF/MAP kinase cascade and Signal Transduction. Gene Ontology (GO) annotations related to this gene include hydrolase microenvironment.

Subsequently, our study found overexpression of ABHD17se activity and serine-type peptidase activity [26]. We then constructed a KPC-ABHD17C-vector/OE cell line stably expressing ABHD17C. In vivo, experiments showed that the KPC-ABHD17C-OE cell line could significantly increase the tumor burden compared with the KPC-ABHD17C-vector group. Our study showed that overexpression of ABHD17C reduced the proportion and function of CD8 + T cells and increased the proportion of CD8 + PD1 + T cells. In addition, the increased expression of ABHD17C promoted the infiltration of MDSCs and further accelerated the formation of an immunosuppressive microenvironment.

Subsequently, our study found that overexpression of ABHD17C can be enriched to glycolysis-related signaling pathways; and then, our results revealed that the immunosuppressive state caused by high expression of ABHD17C was eliminated by knocking down the expression of LDHA [27], which is generally considered to be an important regulator of metabolic processes [28]. Immune checkpoint inhibition is showing promising results in various solid tumors and hematological malignancies as an emerging therapeutic option in cancer. However, PDAC does not respond well to immune checkpoint inhibitors anti-programmed cell death protein 1 (PD-1) [29, 30].

It is well known that the metabolism of tumor cells is positively correlated with immunotherapy resistance[31], and the “metabolic tolerance effect “ of tumor cells can easily reduce the effect of anti-PD1 therapy[32]. In addition, previous studies have shown that there are significant differences in the response of high and low metabolic states to PD1 treatment [20, 30].

With the increasing popularity of tumoral molecules for targeted therapy [33], we also predict the sensitivity of different PDAC patients to immune checkpoint therapy according to the different tumor metabolism caused by the expression of ABHD17C, to improve the treatment efficiency more accurately. Through public databases and drug sensitivity analysis, we first determined that high expression of ABHD17C could indeed predict resistance to anti-PD1 therapy. Subsequently, in vivo, experiments also demonstrated that inhibition of ABHD17C expression could sensitize the sensitivity of anti-PD1 therapy, significantly reduce tumor burden and promote the infiltration of effector T cells.

## Conclusion

In this part of the study, we found that a single marker, ABHD17C, could predict immune checkpoint inhibition therapy by changing the tumor microenvironment, which can achieve the purpose of precise stratified targeted therapy for different patients, and play a guiding role in more accurate typing of tumor patients in the future. However, longitudinal clinical trials should be conducted to verify this hypothesis.

## Supplementary Information

Additional file 1—Supplementary Fig. 1 The KEGG and GO enrichment analysis was conducted with genes which were positively correlated with ABHD17C. a We performed differential gene expression analysis on malignant ductal cell populations from single cell sequencing of pancreatic ductal adenocarcinoma, marking cells with high and low expression of ABHD17C. Red circles indicate high expression, while blue circles indicate low expression; b The GO analysis was conducted for further validating the function of ABHD17C; c The KEGG analysis was conducted for further validating the function of ABHD17C; We filtered significantly differentially expressed genes based on the logFC, and the adj.p_value < 0.05 was considered to represent a significant difference. In supplementary Fig. 1b, c, the adjusted p-values refer to the new p-values obtained after multiple comparison correction was applied to the original p-values in KEGG and GO analyses. The R package clusterProfiler automatically used the FDR method to correct the p-values and identify significant pathways for visualization.

Additional file 2—Supplementary Fig. 2 The immune infiltration analysis was conducted with genes which were positively correlated with ABHD17C. a The immune infiltration analysis was performed with TCGA and GEO datasets by using Cibersort software.

Additional file 3—Supplementary Fig. 3 Data related to Fig.2. a The average expression of ABHD17C in pan-cancer level from TCGA database, particularly in PDAC. Red bar indicates tumor tissue; blue bar indicates normal tissue; b The K-M analysis of public data in the TCGA database was conducted in patients with high ABHD17C expression compared with patients with low ABHD17C expression; disease-free survivaltime was calculated

Additional file 4—Supplementary Fig. 4 Data related to Fig. 6. a the murine KPC-ABHD17C-vector/OE-LDHA-vector/KD cell lines were injected subcutaneously into C57/BL mouse; the subcutaneous tumors were randomly divided into KPC-ABHD17C-vector/OE -vector group and KPC-ABHD17C-vector/OE-LDHA-KD group; Tumor volumes were measured twice a week. The growth curve was plotted according to the size of the tumor two times a week. b The tumors were harvested at the endpoint, weighed, and visualized in the form of a bar chart. Paired student’s t test were performed for in vitro assays and unpaired student’s t test were conducted for in vivo assays. n.s., no significant statistical difference; *p<0.05; **p<0.01; ***p<0.001; ****p<0.0001. In Supplementary Fig. 4a, the tumor volume was calculated based on the long and short diameters measured at multiple time points for both the control and experimental groups. The Benjamini-Hochberg method was used for multiple p-value correction.

Additional file5—Supplementary Fig 5 The ROC analysis and drug sensitivity analysis were performed in anti-PD1 therapy cohorts according to the expression of ABHD17C. a–c The ROC analysis was performed and AUC value was calculated in anti-PD1 therapy cohorts according to the expression of ABHD17C; d the estimation of drug sensitivity in patients with high expression of ABHD17C was conducted among TCGA and GEO datasets.

Additional file 6—Supplementary Fig. 6 Data related to Fig. 7. a–d Immunofluorescence staining of TUNEL and Ki67 in subcutaneous tumor. Representative images were shown. The percentage of Ki67+ and TUNEL+ tumor cells were analyzed by Image J software. Paired student’s t test were performed for in vitro assays and unpaired student’s t test were conducted for in vivo assays. n.s., no significant statistical difference; *p<0.05; **p<0.01; ***p<0.001; ****p<0.0001.

Additional file 7

Additional file 8

Additional file 9

Additional file 10

## Data Availability

The datasets used and/or analyzed during the current study are available from the corresponding author upon reasonable request.
