# Supplementary figures and images for: ABHD17C, a metabolic and immune-related gene signature, predicts prognosis and anti-PD1 therapy response in pancreatic cancer
Source: Discov Oncol. 2023 Jun 5;14:87. doi: 10.1007/s12672-023-00690-7 (PMC10241759; doi:10.1007/s12672-023-00690-7)

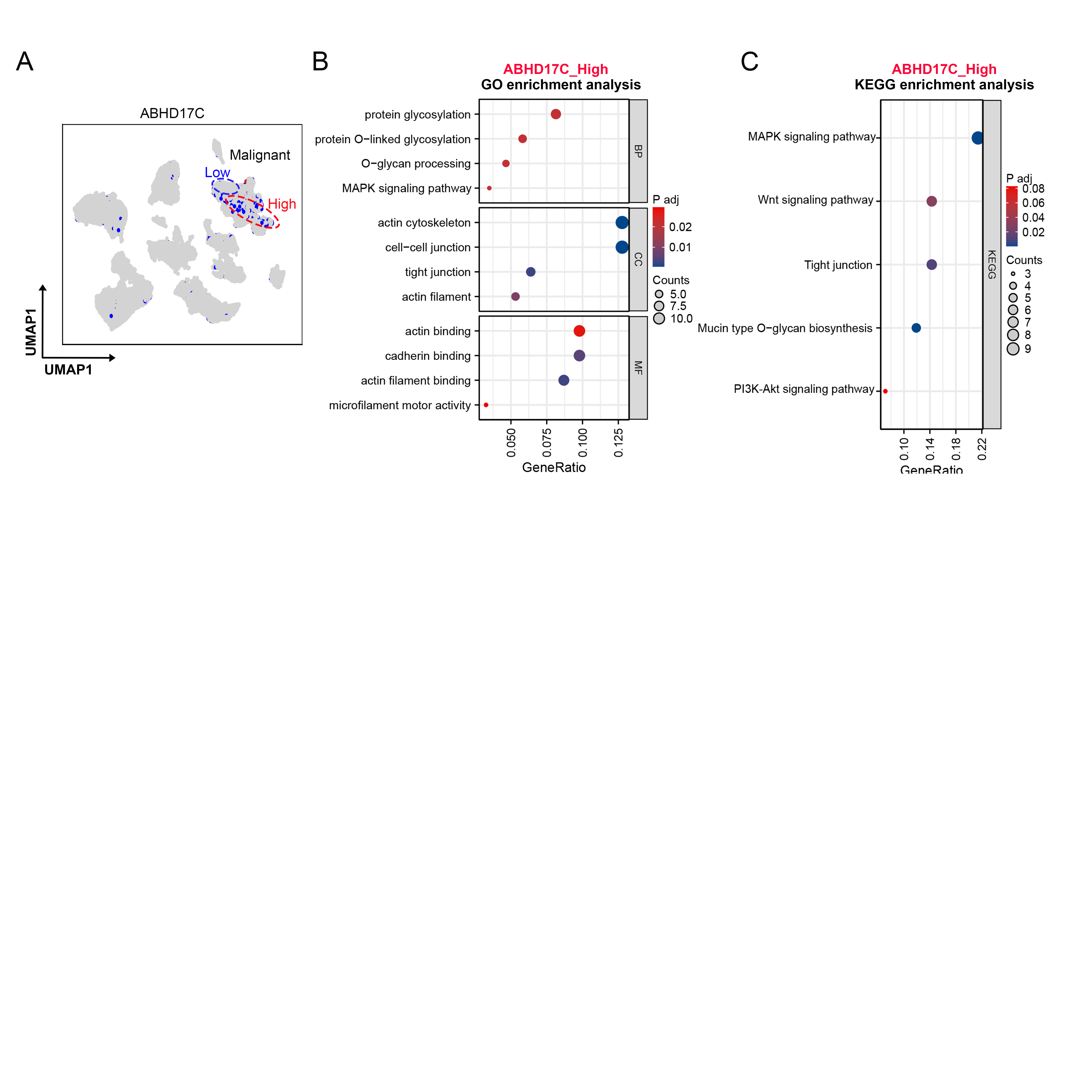

Supplement: Supplementary file 1 — Additional file 1—Supplementary Fig. 1 The KEGG and GO enrichment analysis was conducted with genes which were positively correlated with ABHD17C. a We performed differential gene expression analysis on malignant ductal cell populations from single cell sequencing of pancreatic ductal adenocarcinoma, marking cells with high and low expression of ABHD17C. Red circles indicate high expression, while blue circles indicate low expression; b The GO analysis was conducted for further validating the function of ABHD17C; c The KEGG analysis was conducted for further validating the function of ABHD17C; We filtered significantly differentially expressed genes based on the logFC, and the adj.p_value < 0.05 was considered to represent a significant difference. In supplementary Fig. 1b, c, the adjusted p-values refer to the new p-values obtained after multiple comparison correction was applied to the original p-values in KEGG and GO analyses. The R package clusterProfiler automatically used the FDR method to correct the p-values and identify significant pathways for visualization. [file 12672_2023_690_MOESM1_ESM.tif]

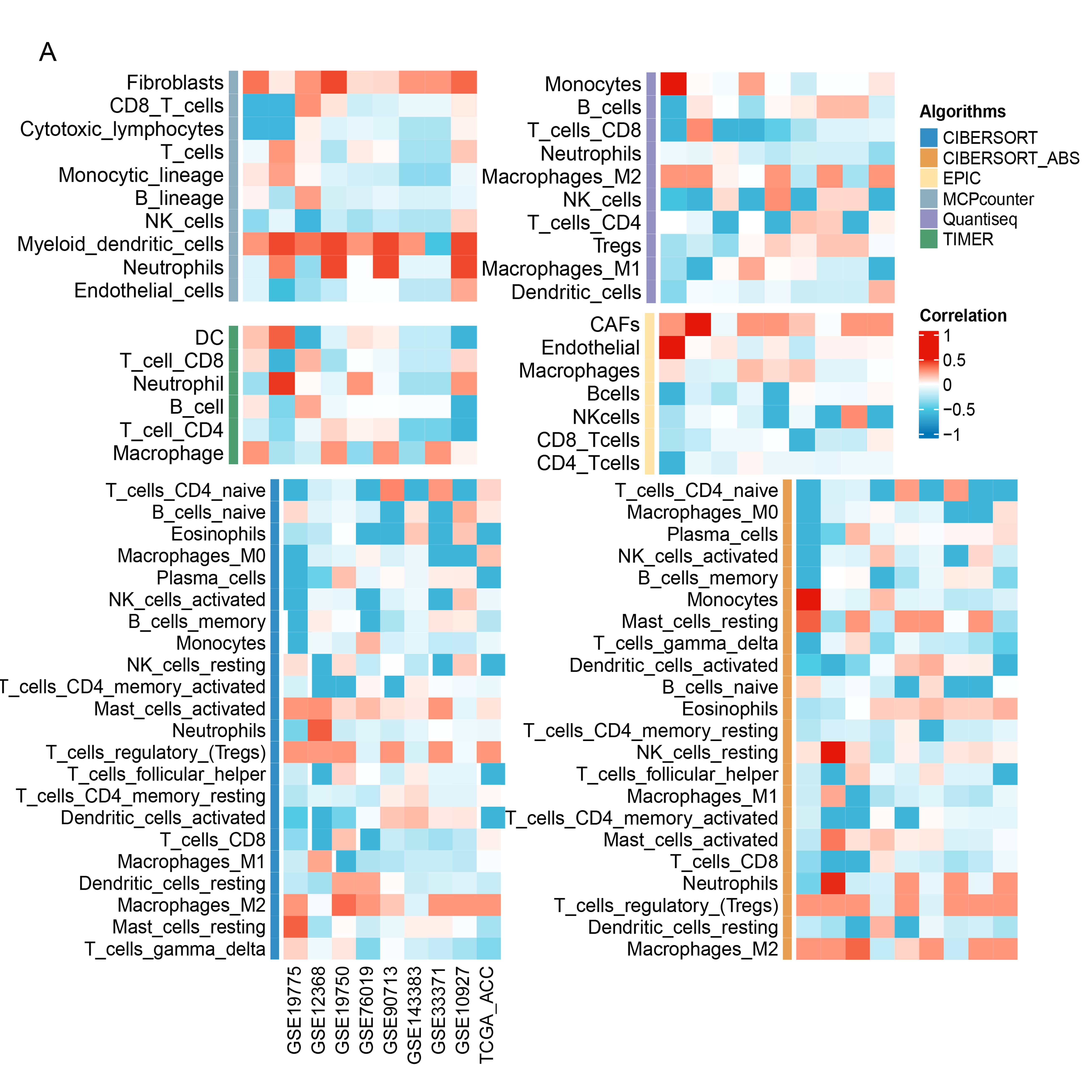

Supplement: Supplementary file 2 — Additional file 2—Supplementary Fig. 2 The immune infiltration analysis was conducted with genes which were positively correlated with ABHD17C. a The immune infiltration analysis was performed with TCGA and GEO datasets by using Cibersort software. [file 12672_2023_690_MOESM2_ESM.tif]

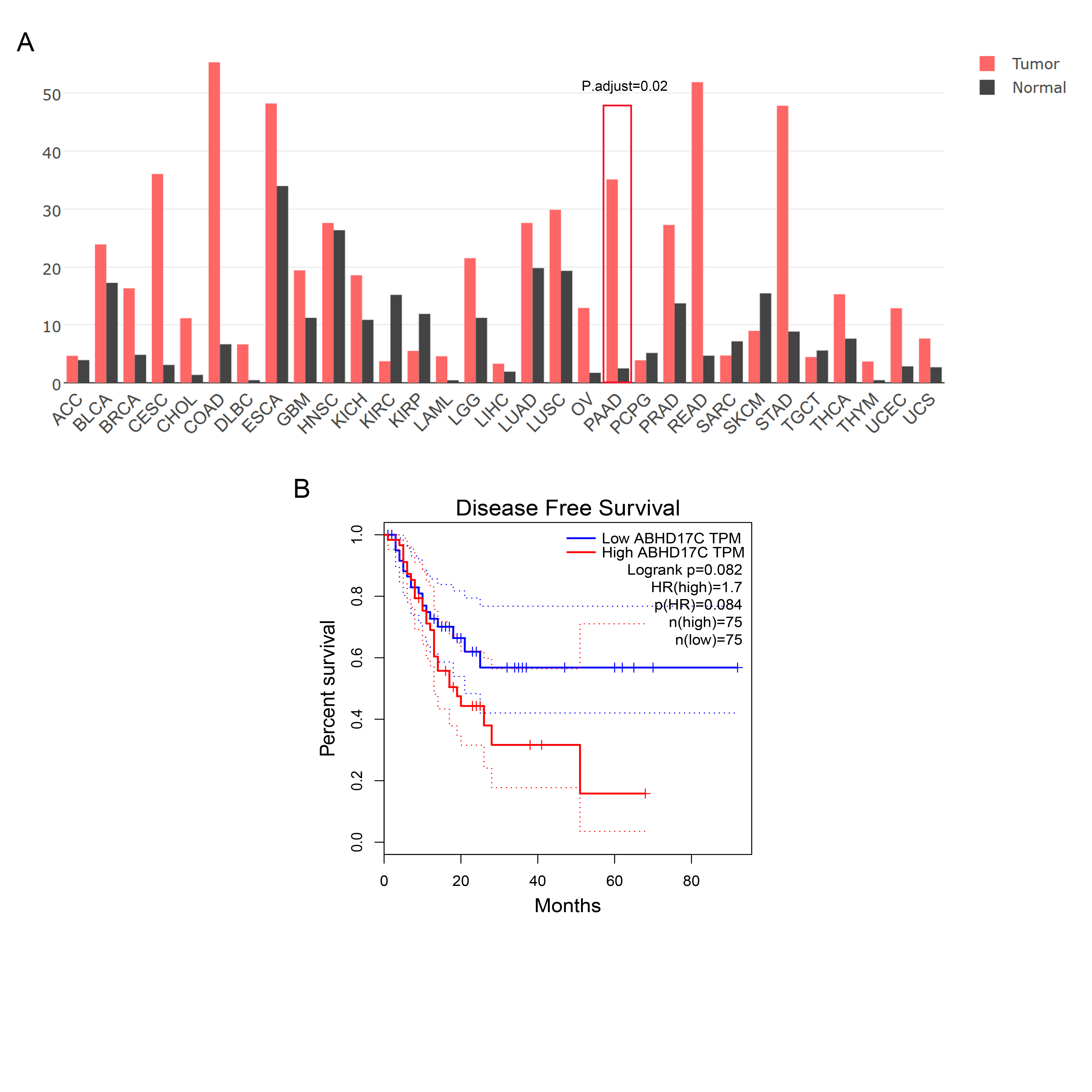

Supplement: Supplementary file 3 — Additional file 3—Supplementary Fig. 3 Data related to Fig.2. a The average expression of ABHD17C in pan-cancer level from TCGA database, particularly in PDAC. Red bar indicates tumor tissue; blue bar indicates normal tissue; b The K-M analysis of public data in the TCGA database was conducted in patients with high ABHD17C expression compared with patients with low ABHD17C expression; disease-free survivaltime was calculated [file 12672_2023_690_MOESM3_ESM.tif]

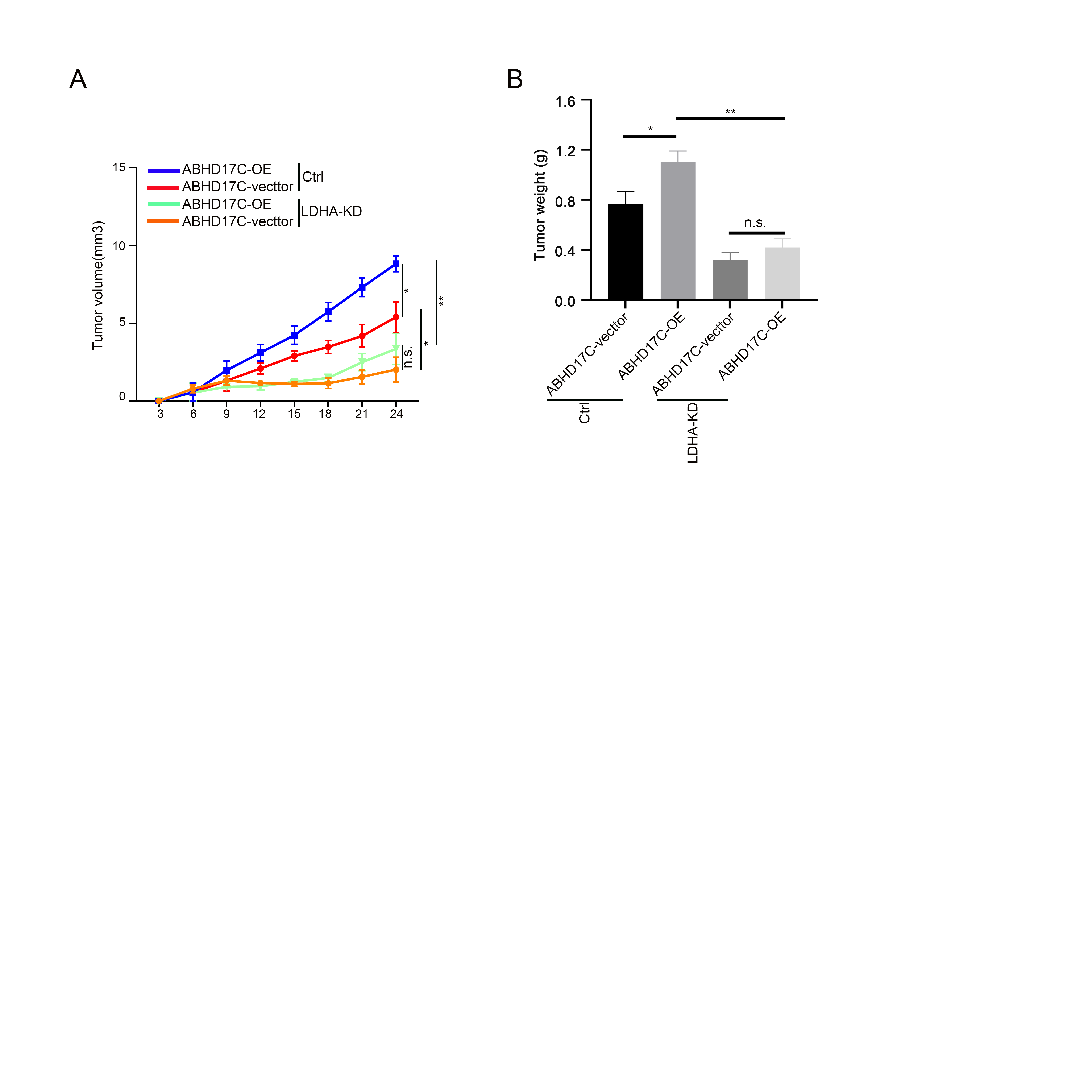

Supplement: Supplementary file 4 — Additional file 4—Supplementary Fig. 4 Data related to Fig. 6. a the murine KPC-ABHD17C-vector/OE-LDHA-vector/KD cell lines were injected subcutaneously into C57/BL mouse; the subcutaneous tumors were randomly divided into KPC-ABHD17C-vector/OE -vector group and KPC-ABHD17C-vector/OE-LDHA-KD group; Tumor volumes were measured twice a week. The growth curve was plotted according to the size of the tumor two times a week. b The tumors were harvested at the endpoint, weighed, and visualized in the form of a bar chart. Paired student’s t test were performed for in vitro assays and unpaired student’s t test were conducted for in vivo assays. n.s., no significant statistical difference; *p<0.05; **p<0.01; ***p<0.001; ****p<0.0001. In Supplementary Fig. 4a, the tumor volume was calculated based on the long and short diameters measured at multiple time points for both the control and experimental groups. The Benjamini-Hochberg method was used for multiple p-value correction. [file 12672_2023_690_MOESM4_ESM.tif]

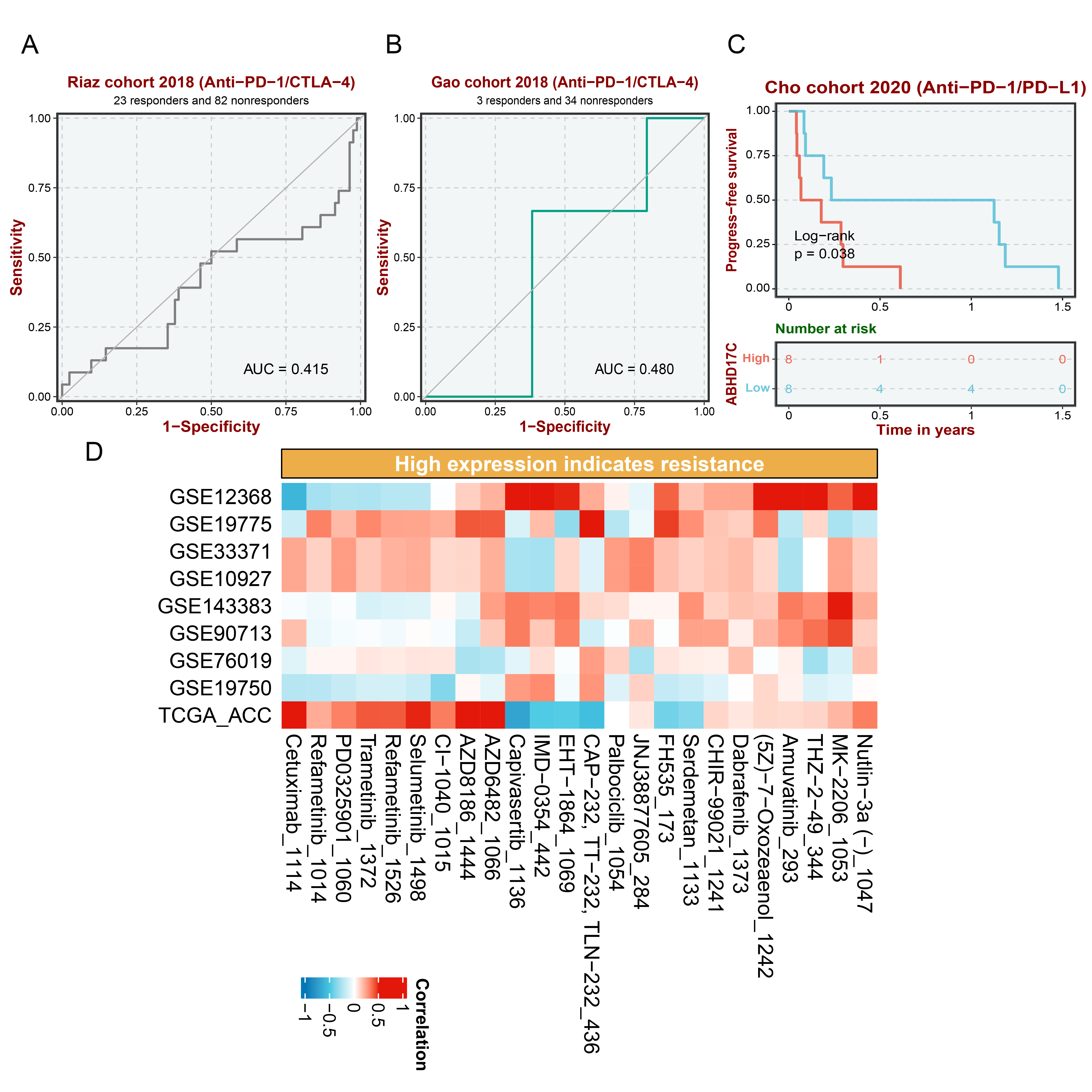

Supplement: Supplementary file 5 — Additional file5—Supplementary Fig 5 The ROC analysis and drug sensitivity analysis were performed in anti-PD1 therapy cohorts according to the expression of ABHD17C. a–c The ROC analysis was performed and AUC value was calculated in anti-PD1 therapy cohorts according to the expression of ABHD17C; d the estimation of drug sensitivity in patients with high expression of ABHD17C was conducted among TCGA and GEO datasets. [file 12672_2023_690_MOESM5_ESM.tif]

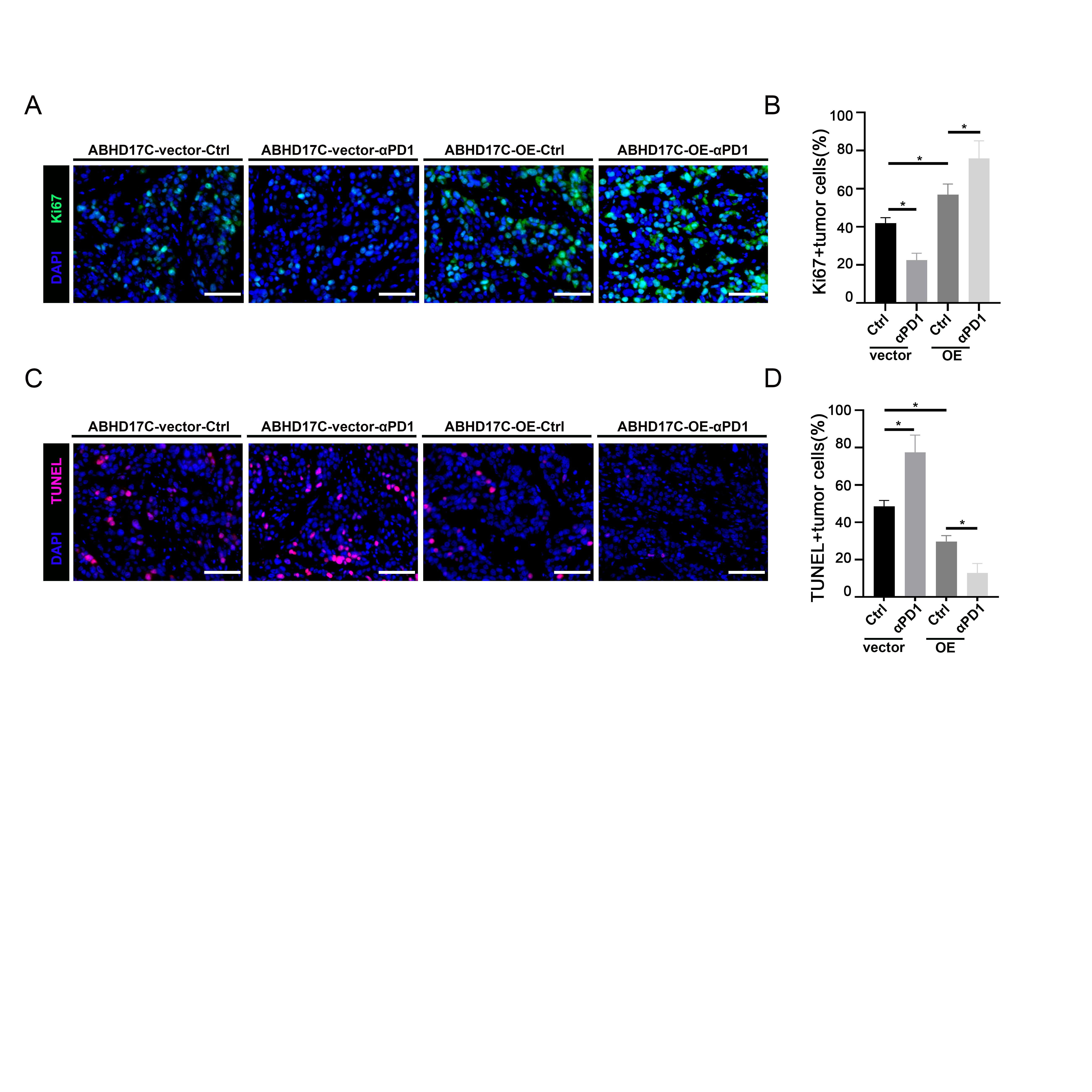

Supplement: Supplementary file 6 — Additional file 6—Supplementary Fig. 6 Data related to Fig. 7. a–d Immunofluorescence staining of TUNEL and Ki67 in subcutaneous tumor. Representative images were shown. The percentage of Ki67+ and TUNEL+ tumor cells were analyzed by Image J software. Paired student’s t test were performed for in vitro assays and unpaired student’s t test were conducted for in vivo assays. n.s., no significant statistical difference; *p<0.05; **p<0.01; ***p<0.001; ****p<0.0001. [file 12672_2023_690_MOESM6_ESM.tif]
