# Supplementary material for: ABHD17C, a metabolic and immune-related gene signature, predicts prognosis and anti-PD1 therapy response in pancreatic cancer
Source: Discov Oncol. 2023 Jun 5;14:87. doi: 10.1007/s12672-023-00690-7 (PMC10241759; doi:10.1007/s12672-023-00690-7)
Supplement: Supplementary file 7 — Additional file 7 [file 12672_2023_690_MOESM7_ESM.docx]

**Supplementary tables**

**Supplementary table1:** The detailed information of the mRNA sequencing of patients from our center was shown in a supplementary EXCEL table named “Table S1 Detailed information of mRNA sequencing”

**Supplementary table2:** The detailed information of the metabolic-immune-related genes(M-I-DEGs) was shown in a supplementary EXCEL table named “Table S2 Detailed information of M-I-DEGs”

**Supplementary table3: Correlation of ABHD17C expression to clinicopathological features in PDAC.**

|  |  | **ABHD17C(n)^b^** | |  |  |  |
| --- | --- | --- | --- | --- | --- | --- |
| **Parameters** |  | **low** | **high** | **χ^2^** | ***P*** | **r** |
| **Age(years)** | <60 | 24 | 27 | 1.300 | 0.367 | 0.108 |
|  | ≥60 | 15 | 28 |  |  |  |
| **Gender** | Male | 25 | 34 | 0.000 | 0.896 | 0.000 |
|  | Female | 14 | 21 |  |  |  |
| **Histological grade** | G1, G2 | 31 | 32 | 4.205 | **0.031^a^** | 0.226 |
|  | G3 | 7 | 24 |  |  |  |
| **p TNM stage** | IA, IB | 30 | 27 | 5.3 | **0.034^a^** | 0.205 |
|  | IIA, IIB | 11 | 26 |  |  |  |
| **Tumor size** | T1≤3.5cm | 35 | 18 | 15.54 | **0.000^a^** | 0.520 |
|  | T2>3.5cm | 8 | 33 |  |  |  |
| **LN metastasis** | N0 | 34 | 40 | 0.285 | 0.762 | -0.044 |
|  | N1 | 12 | 8 |  |  |  |

Note: Data was based on IHC assay. Statistical data on ABHD17C expression in relation to clinic- histopathologic features for surgical PDAC specimens. *P* values were calculated using the chi-square test. ^a^ Statistically significant (*P*<0.05). ^b^ Here ABHD17C expression was divided into high-ABHD17C and low-ABHD17C according to staining scores.

**Supplementary table4: Analysis of ABHD17C Expression and Clinical Pathological Features in Pancreatic Cancer: A Univariate and Multivariate Study using the TCGA Database**

| Characteristics | Total(N) | Univariate analysis | |  | Multivariate analysis | |
| --- | --- | --- | --- | --- | --- | --- |
|  |  | Hazard ratio (95% CI) | P value |  | Hazard ratio (95% CI) | P value |
| Pathologic N stage | 174 |  | **0.002** |  |  |  |
| N0 | 50 | Reference |  |  | Reference |  |
| N1 | 124 | 2.161 (1.287 - 3.627) | **0.004** |  | 1.860 (0.831 - 4.165) | **0.0231** |
| Pathologic stage | 168 |  | **0.016** |  |  |  |
| Stage I | 21 | Reference |  |  | Reference |  |
| Stage II | 147 | 2.342 (1.074 - 5.109) | **0.032** |  | 0.937 (0.305 - 2.875) | 0.910 |
| Gender | 179 |  | 0.320 |  |  |  |
| Female | 80 | Reference |  |  |  |  |
| Male | 99 | 0.813 (0.541 - 1.222) | 0.319 |  |  |  |
| Age | 179 |  | 0.230 |  |  |  |
| <= 65 | 94 | Reference |  |  |  |  |
| > 65 | 85 | 1.285 (0.853 - 1.937) | 0.230 |  |  |  |
| Histologic grade | 177 |  | 0.060 |  |  |  |
| G1&G2 | 127 | Reference |  |  | Reference |  |
| G3&G4 | 50 | 1.532 (0.993 - 2.363) | 0.054 |  | 1.503 (0.855 - 2.644) | **0.0457** |
| Residual tumor | 160 |  | **0.021** |  |  |  |
| R0 | 107 | Reference |  |  | Reference |  |
| R1 | 53 | 1.713 (1.096 - 2.678) | **0.018** |  | 1.470 (0.841 - 2.570) | **0.0476** |
| Primary therapy outcome | 140 |  | **< 0.001** |  |  |  |
| PD&SD | 59 | Reference |  |  | Reference |  |
| PR&CR | 81 | 0.425 (0.267 - 0.675) | **< 0.001** |  | 0.548 (0.326 - 0.922) | **0.023** |
| ABHD17C | 179 |  | **0.002** |  |  |  |
| Low | 89 | Reference |  |  | Reference |  |
| High | 90 | 1.889 (1.243 - 2.870) | **0.003** |  | 1.362 (0.780 - 2.380) | **0.0478** |

Univariate cox regression analysis and Multivariate cox regression analysis among all clinical characteristic features and the expression of ABHD17C were performed in pancreatic cancer dataset from TCGA database. Black colored indicate that the variate is statistically significant (*P*<0.05).

**Supplementary table5:** Pathological Features, Subtyping, and Prognostic Information of Pancreatic Ductal Adenocarcinoma (PDAC) Patients in TCGA Database was shown in a supplementary EXCEL table named “Table S5 Detailed information of Pathological Features and Prognostic Information of PDAC”
